# DRP1 downregulation impairs mitophagy, driving mitochondrial ROS and SASP production in rheumatoid arthritis CD4^+^PD-1^+^T cells

**DOI:** 10.1016/j.redox.2025.103818

**Published:** 2025-08-09

**Authors:** Ziran Bai, Jinyi Ren, Jiaqing Liu, Cheng Zhang, Huina Huang, Xiangge Zhao, Xianmei Chen, Jing Wei, Jingjing Qi, Siwen Yang, Weiping Li, Yawei Tang, Guan Wang, Xia Li

**Affiliations:** a Department of Immunology, College of Basic Medical Science, Dalian Medical University, Dalian, Liaoning, China; b Department of Hematology, the Second Hospital of Dalian Medical University, Dalian, Liaoning, China; c Department of Flow Cytometry Center, the Second Hospital of Dalian Medical University, Dalian, Liaoning, China

**Keywords:** Rheumatoid arthritis, CD4^+^PD-1^+^T cells, Senescence-associated secretory phenotype, Dynamin-related protein 1, Mitochondrial reactive oxygen species

## Abstract

T cell senescence occurs in patients with rheumatoid arthritis (RA), but the specific phenotype and its contribution to tissue-destructive inflammation remain unclear. Here, we aim to investigate whether PD-1 marks pathogenic senescent CD4^+^T cells and to explore the role and mechanism of senescent CD4^+^PD-1^+^T cells in RA pathogenesis. Here, we identified an expanded population of CD4^+^PD-1^+^T cells in RA patients that exhibited hallmark senescence features, including elevated senescence-associated secretory phenotype (SASP) production. Adoptive transfer experiments demonstrated that CD4^+^PD-1^+^T cells significantly accelerated disease progression in collagen-induced arthritis (CIA) models. Mechanistically, we demonstrated that RA CD4^+^PD-1^+^T cells showed decreased expression of dynamin-related protein 1 (DRP1) and impaired mitophagy, leading to mitochondrial reactive oxygen species (MtROS) accumulation and subsequent SASP production. Importantly, PD-1 signaling transcriptionally suppressed DRP1 expression through hypoxia inducible factor 1 alpha subunit (HIF-1α) inhibition. Our findings establish CD4^+^PD-1^+^T cells as a pathogenic senescent subset that drives RA progression through a PD-1-DRP1-mitophagy-SASP axis.

## Graphical abstract

**Figure:**
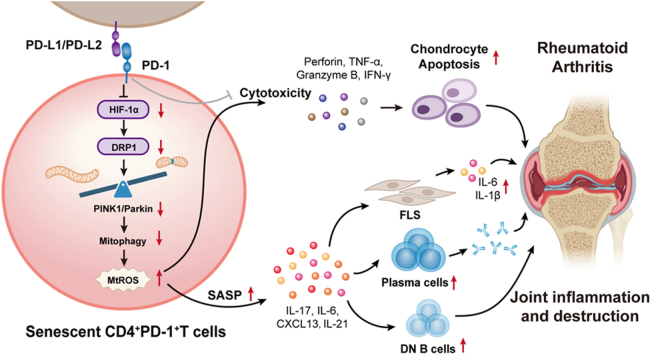

## 1 Introduction

Rheumatoid arthritis (RA) is a multifactorial autoimmune disease characterized by joint inflammation and persistent synovitis, ultimately leading to cartilage and bone destruction [[1], [2], [3]]. Dysregulated CD4^+^T cell responses play a central role in RA pathogenesis, and targeting pathogenic T cell subsets has emerged as a promising therapeutic strategy [4,5]. Notably, CD4^+^T cells in RA display premature senescence features, including a senescence-associated secretory phenotype (SASP) and cytotoxic effector functions, which perpetuate inflammation and exacerbate disease progression [[6], [7], [8], [9], [10]].

It is well established that senescent T cells, particularly those in the effector memory (EM) subset, exhibit a unique phenotype characterized by the loss of co-stimulatory molecules such as CD27 and CD28, and the expression of natural killer cell receptors, including killer cell lectin-like receptor G1 (KLRG-1) and CD57 [[11], [12], [13]]. A recent study reported that increased expression of programmed death receptor 1 (PD-1) on T cells has been reported with aging [[14], [15], [16]]. Importantly, CD4^+^T cells expressing PD-1 are expanded in the peripheral blood in RA patients [[17], [18], [19]]. However, the significance of PD-1 as a marker of senescent CD4^+^T cells and its unique role in RA remains to be fully elucidated.

Mitochondrial dysfunction is a hallmark of cell senescence, characterized by reduced respiratory capacity, decreased mitochondrial membrane potential (MMP), typically accompanied by increased production of reactive oxygen species (ROS) [[20], [21], [22]]. Imbalanced mitochondrial dynamics and impaired mitophagy lead to accumulation of dysfunctional mitochondria [23,24]. The translocation of dynamin-related protein 1 (DRP1) is a key factor for mitochondrial homeostasis, mediating mitochondrial fission and mitophagy [25]. While reduced DRP1 expression has been observed in T cells from systemic lupus erythematosus (SLE) patients and related to disease development in lupus-prone mice [26], the role of mitochondrial dynamics and mitochondrial dysfunction in RA-related T cell senescence is yet to be determined.

This study aims to identify CD4^+^PD-1^+^T cells as the pivotal senescent T cell subset in RA, investigate their role in RA pathogenesis, and further elucidate the mechanistic contributions of mitochondrial dysfunction and PD-1 signaling to the senescence of CD4^+^PD-1^+^T cells. Our findings unveil a PD-1–mitochondria–senescence axis in CD4^+^T cells, offering novel therapeutic strategies to target pathogenic senescent T cells in RA and redefining the functional role of PD-1 signaling in RA pathophysiology.

## 2 Materials and methods

### 2.1 Human blood sample

Peripheral blood samples of RA patients who fulfilled the American College of Rheumatology criteria for RA in the Department of Rheumatology (ACR1987) were obtained from the Department of Rheumatology and Immunology of the Second Affiliated Hospital of Dalian Medical University in China. The age- and sex-matched health controls (HC) were obtained from the medical examination center in this hospital. The study protocol was approved by the ethics committee of the Second Hospital of Dalian Medical University (Approval No: 2023-253), and informed consent was obtained from all subjects. Detailed clinical characteristics are shown in Supplemental Table 1.

### 2.2 Cell isolation, purification, and activation

Peripheral blood mononuclear cells (PBMCs) were purified from peripheral blood by Ficoll-density gradient centrifugation. Human CD4^+^T and B cells were purified from PBMCs using the MojoSort™ Human CD4^+^T or Pan B Cell Isolation Kit according to the manufacturer's protocols (BioLegend). The CD4^+^PD-1^+^T and CD4^+^PD-1^−^T cells were isolated by *anti*-PD-1 (PE) and *anti*-PE Nanobeads (BioLegend). The purified RA CD4^+^T cells and PBMCs were activated with anti-CD3 and anti-CD28 antibodies and treated with mitoquinone (MitoQ, 200 nM, Biovision), mdivi-1 (1 μM, Selleck), Drpitor1a (1 μM, MCE), MYLS22 (10 μg/mL, MCE), or CCCP (1 μM, Sigma-Aldrich) for 24–72 h. For the PD-1 Ligation assay, plates were coated with anti-CD3 (5 μg/mL, Invitrogen) and with either PD-L1, PD-L2, or immunoglobulin (Ig) G1 (10 μg/mL, Biolegend) for 2 h at 37 °C and 5 % CO_2_. Then, the purified RA CD4^+^PD-1^+^T cells were plated in a final volume of 200 μL of culture medium containing anti-CD28 (2 μg/mL, Invitrogen) for 24–72 h.

### 2.3 Flow cytometry

Cell surface staining was performed using BioLegend or eBioscience reagents for 20 min. For intracellular and nuclear staining, cells were fixed and permeabilized with an intracellular fixation and permeabilization buffer set (eBioscience) for 1 h, followed by staining with intracellular nuclear antibodies for 30 min. For cytokine staining, the cells were treated with phorbol myristate acetate (PMA) (50 ng/mL) and ionomycin (1 μg/mL) for 4 h in the presence of brefeldin A (10 μg/mL). The antibodies for staining are shown in Supplementary Table 2. All stained cells were analyzed on the Flow Cytometer (NovoCyte 2060R), and data were analyzed with NovoExpress software.

### 2.4 Cell proliferation assay

Cell proliferation was stained using the Carboxyfluorescein succinimidyl amino ester (CFSE) Cell Labeling Assay (eBioscience). CFSE was added at a final concentration of 1.5 μM to label RA CD4^+^PD-1^+^T cells and RA CD4^+^PD-1^−^T cells. The CFSE-labeled cells were then incubated in RPMI-1640 complete medium containing 10 % FBS in a 37 °C, 5 % CO_2_ incubator for 5 days. Then analyzed using flow cytometry.

### 2.5 Senescence-associated β-galactosidase (SA-β-Gal) staining

Cells were resuspended in 100 μL of fresh media containing 0.3 μL of Senescence Dye (Abcam) and incubated for 1 h at 37 °C, 5 % CO_2_. Subsequently, the cells were washed with 500 μL 1 × Wash buffer and surface-stained with anti-CD4 and *anti*-PD-1 antibodies. Afterward, cells were permeabilized and stained with *anti*-Ki67 antibody for 30 min. Flow cytometric analysis was performed promptly following the staining procedures.

### 2.6 Quantitative Polymerase Chain Reaction (qPCR)

Total RNA was extracted from RA-CD4^+^T cells using RNAex Pro Reagent (AG, China), and cDNA was transcribed using 5 × All-In-One RT MasterMix (Abm), both according to the manufacturer's instructions. Real-time PCR was carried out with the Seven Fast Two Steps RT & qPCR kit (SEVEN, China) on the CFX96 Real-Time PCR Detection System (Bio-Rad). Gene expression was normalized to the relative quantity of β-actin gene expression and quantified by standard 2^−ΔΔCT^ calculation. Primer sequences are listed in Supplemental Table 3.

### 2.7 Measurement of mitochondrial reactive oxygen species (MtROS) levels

MtROS levels were assessed by incubating cells with 5 μM MitoSOX Red probe (Invitrogen) for 30 min at 37 °C. Then wash the cells gently three times with warm Hank's and analyze by flow cytometry.

### 2.8 JC-10 staining

Mitochondrial membrane potential in T cells was determined by Mitochondrial Membrane Potential Kit (JC-10 assay; Solarbio, China) according to the instructions of the manufacturer. Briefly, Cells were stained with JC-10 staining solution at 37 °C for 20 min. Then wash the cells gently three times with warm Hank's and analyze by flow cytometry.

### 2.9 Mitotracker Green staining

Cells were stained with 10 nmol/L Mitotracker Green (Beyotime, China) at 37 °C for 30 min. Then the cells were imaged using an Olympus microscope or analyzed by flow cytometry. The mitochondrial aspect ratio (length/width) was quantified using ImageJ software.

### 2.10 Mitophagy measurement

The mitophagy flux in RA CD4^+^PD-1^+^T cells and CD4^+^PD-1^−^T cells was detected by using a mitophagy reporter, mt-mKeima (Hanbio Technology, China). The mt-Keima sequence is inserted into an adenoviral vector to achieve efficient cellular infection. By observing fluorescence intensity under different excitation wavelengths using fluorescence microscopy, the status of mitophagy within cells can be determined. Briefly, RA CD4^+^PD-1^+^T cells and CD4^+^PD-1^−^T cells were isolated by Immunomagnetic Beads. A density of 1.5 × 10^5^ cells per well was cultured overnight in a 37 °C, 5 % CO_2_ incubator. The following day, cells were infected with the adenovirus. After 6–8 h, the culture medium was refreshed to support continued cellular growth. Fluorescence was then observed 72 h post-infection using 440 nm and 550 nm laser excitation. A significant increase in fluorescence under 550 nm (red) excitation, coupled with a decrease under 440 nm (green) excitation, indicated enhanced mitophagy. For flow cytometry analysis, cells were treated as described above, and the proportion of red-positive cells (550 nm, mt-keima pH 4) was determined [27,28].

### 2.11 Western blot

Cells were dissolved in RIPA lysis buffer (Beyotime, China). The protein was separated by sodium dodecyl sulfate-polyacrylamide gel electrophoresis (SDS-PAGE) and electrophoretically transferred to nitrocellulose filter membranes. Membranes were incubated with antibodies, including GAPDH (Cell Signaling Technology), DRP1 (Proteintech, China), PINK1 (Cell Signaling Technology), Parkin (Cell Signaling Technology), P53 (Cohesion), P16 (Cohesion), and P21 (Affinity), then incubated with a fluorescent secondary antibody (LICOR). The Odyssey CLx Infrared Scanner was utilized to detect the results.

### 2.12 Cell coculture

For T-B cell coculture, sorted RA CD4^+^PD-1^+^T cells and RA CD4^+^PD-1^−^T cells were co-cultured with HC Pan B cells at a ratio of 1:2 in 200 μL RPMI-1640/10 % FBS and stimulated with SEB (1 μg/mL, Toxin Technology Inc.) for 7 days. The proportion of CD19^+^B cell subsets was detected by flow cytometry. For T cells-RA fibroblast-like synoviocytes (FLS) co-culture, sorted RA CD4^+^PD-1^+^T cells and RA CD4^+^PD-1^−^T cells stimulated with anti-CD3 and anti-CD28 were respectively co-cultured with RA-FLS at a ratio of 5:1 in 1 mL DMEM/10 % FBS for 48 h. After removing suspended CD4^+^T cells by PBS washing, total RNA was extracted from adherent RA-FLS and analyzed via qPCR for *IL-1β* and *IL-6* expression. For T cells-chondrocyte co-culture, sorted RA CD4^+^PD-1^+^T cells and RA CD4^+^PD-1^−^T cells stimulated with anti-CD3 and anti-CD28 were respectively co-cultured with osteoarthritis (OA) chondrocytes at a ratio of 5:1 in 1 mL DMEM/10 % FBS for 48 h. The apoptosis of adherent OA chondrocytes was detected by flow cytometry.

### 2.13 Cell transfection

To knock down DRP1 and Parkin expression: Small interfering RNAs (siRNAs) targeting DRP1 or Parkin, along with a non-targeting negative control (GenePharma, China), were transiently transfected into Jurkat cells using Lipofectamine 3000 (Invitrogen) in serum-free medium at the manufacturer's recommended working concentration. For DRP1 overexpression: The pcDNA3.1 vector containing the human DRP1 gene sequence (pcDNA3.1-DRP1) and the negative control pcDNA3.1-NC were constructed and provided by GenePharma. Then Jurkat cells were transfected with Lipofectamine 3000 according to the manufacturer's instructions. Gene expression was analyzed by qPCR (24 h post-transfection) and Western blot (48 h post-transfection) for mRNA and protein quantification, respectively.

### 2.14 Collagen-Induced Arthritis (CIA) model

Male DBA/1J mice aged 6–8 weeks were purchased from Shanghai SLAC Laboratory Animals Company and housed under pathogen-free conditions at the Laboratory Animal Center of Dalian Medical University. For the primary immunization, mice were subcutaneously injected in the tail with the emulsion formed by type II bovine collagen (Chondrex, USA) and Complete Freund adjuvant (CFA, Chondrex). Administer a booster injection consisting of type II bovine collagen and Incomplete Freund adjuvant (IFA, Chondrex) emulsion on day 21. The injection consisted of 100 μL of an emulsion containing 100 μg of collagen and 2 mg/mL of CFA or IFA. The mice's paw thickness and disease activity score were assessed. Disease activity scores were derived from the evaluation of clinical arthritis in all four limbs as reported by the scoring system for the evaluation of arthritis severity. The scoring system was defined as 0, no evidence of erythema and swelling; 1, erythema and mild swelling confined to the tarsals or ankle joint; 2, erythema and mild swelling extending from the ankle to the tarsals; 3, erythema and moderate swelling extending from the ankle to the metatarsal joints; and 4, erythema and severe swelling encompassing the ankle, foot and digits or ankylosis of the limb. All experiments complied with the guidelines established by the Institutional Animal Care and Use Committee (IACUC). The study was approved by the Ethics Committee of Dalian Medical University (Approval No: AEE24013).

### 2.15 Adoptive transfer

CD4^+^PD-1^−^T cells and CD4^+^PD-1^+^T cells were purified from the CIA mice's spleen. 1 × 10^6^ CD4^+^PD-1^−^T cells, CD4^+^PD-1^+^T cells, MitoQ-treated CD4^+^PD-1^+^T cells, or mdivi-1-treated CD4^+^PD-1^+^T cells and CD4^+^PD-1^−^T cells were respectively transferred into CIA mice at day 21 via tail vein injection. The study covered the healthy control mice group (n = 4, per experiments), the CIA mice group (n = 4, per experiments), CIA + CD4^+^PD-1^+^T cells mice group (n = 4 or n = 3), CIA + CD4^+^PD-1^−^T cells mice group (n = 4), CIA + MitoQ-treated CD4^+^PD-1^+^T cells mice group (n = 3), CIA + mdivi-1-treated CD4^+^PD-1^+^T cells (n = 4), CIA + mdivi-1-treated CD4^+^PD-1^+^T cells (n = 4), and CIA + mdivi-1-treated CD4^+^PD-1^−^T cells (n = 4).

### 2.16 Histological analysis and immunohistochemistry staining

The paws of CIA or control mice were fixed in 4 % paraformaldehyde overnight, followed by decalcification in 14 % EDTA and embedding in paraffin. Tissue sections of 5-μm thickness were then generated. The sections were placed in citric acid buffer at 94 °C for 15 min to repair the antigen. An appropriate amount of endogenous peroxidase blocker was added dropwise to the slides for 10 min at room temperature. Next, the sections were blocked with 5 % BSA at room temperature for 30 min, and then H&E and safranin O-fast green staining were performed. The expression of IL-1β and IL-6 in synovium was detected by using immunohistochemistry. Sections were incubated with the corresponding primary antibodies in a humidified chamber overnight. A universal two-step detection kit was used for the secondary processing of tissue samples following the manufacturer's instructions. A DAB stain and hematoxylin counterstain were used. The sections were dehydrated in gradient ethanol, transparentized with xylene, and finally sealed with neutral gel.

### 2.17 Immunofluorescence staining

The synovial tissues of RA and OA patients or mice paws were made into paraffin sections. Then sections were placed in citric acid buffer at 94 °C for 15 min to repair the antigen and were blocked in PBS with 5 % BSA at room temperature for 30 min. To detect CD4^+^PD-1^+^T cells in synovial tissue, the sections were incubated with anti-human/mouse CD4-FITC and anti-human/mouse PD-1-PE in a 37 °C wet box for 1 h. Then, they were sealed with tablets containing DAPI, which was used to stain cellular nuclei, and the images were scanned under fluorescence microscopy. The mean fluorescence intensity (MFI) of PD-1 was analyzed by Image J.

### 2.18 Bioinformatics analysis

GSE199490 was downloaded from the GEO database (http://www.ncbi.nih.gov/geo) and analyzed by the OmicShare (https://www.omicstudio.cn/tool) and the Sangerbox (http://sangerbox.com/home.html) tools.

### 2.19 Statistical analysis

Statistical analysis was performed with GraphPad Prism 9 software. Statistical differences were analyzed by paired *t*-test, unpaired *t*-test, Mann-Whitney test, One-way ANOVA, and Two-way ANOVA. The Pearson test was used to determine the correlation between variables. Data are presented as mean ± standard deviation (SD). The *P*-values <0.05 were considered significant. Asterisks mark the significant differences between different groups (∗, *P* < 0.05; ∗∗, *P* < 0.01; and ∗∗∗, *P* < 0.001).

## 3 Results

### 3.1 CD4^+^PD-1^+^T cells display cellular senescence characteristics

Recent evidence suggests that T cells are highly sensitive to senescence, and senescent CD4^+^T cells are implicated in promoting inflammatory processes that significantly accelerate the RA disease process [7,8]. The Ki67^−^SA-β-Gal^+^ cells are recognized as markers of senescent cells [29]. In our studies, we confirmed that compared with HC, RA CD4^+^T cells displayed more obvious cellular senescence features, characterised by elevated SA-β-Gal activity (CD4^+^Ki67^−^T cells), increased gene expression of senescence-associated cyclin-dependent kinase inhibitors (*P53*, *P21,* and *P16*), and enhanced secretion of SASP factors (Fig. S1A–1D). However, conventional T cell senescence surface markers (CD28^neg^/CD27^neg^/CD57^+^/CCR7^neg^) exhibited no statistically significant differences in CD4^+^T cells between RA patients and HC (Fig. S1E).

**Fig. 1:**
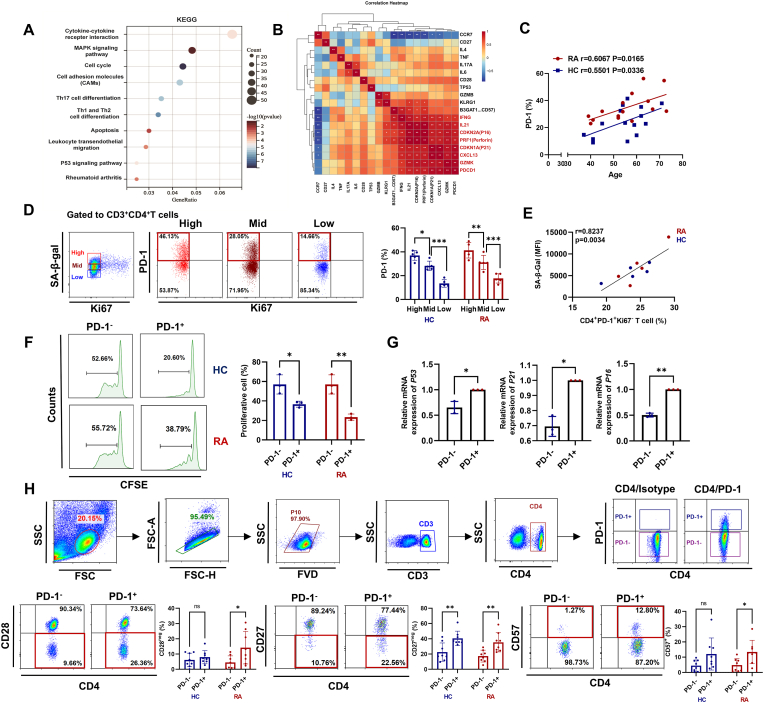
**CD4^+^PD-1^+^T cells display cellular senescence characteristics.** (A) KEGG pathway analysis was used to analyze the gene pathway-related differential expression genes in RA-CD4^+^PD-1^+^T cells and RA-CD4^+^PD-1^−^T cells from the gene set GSE199490. (B) The correlation analysis between PD-1 and T cell senescence-related genes (*GZMK*, *CXCL13*, *P21*, *Perforin*, *IL-21*, *IFNG*) in CD4^+^PD-1^+^T cells from GSE199490. (C) The correlation analysis between the age and proportion of CD4^+^PD-1^+^T cells in the peripheral blood of RA patients (n = 15) and the healthy control (HC) group (n = 15). (D) CD4^+^T cells from RA patients (n = 5) and HC (n = 5) were labeled using the SA-β-Gal probe and divided into three groups: SA-β-Gal^high^, SA-β-Gal^mid^, and SA-β-Gal^low^, then the proportion of Ki67^−^PD-1^+^T was analyzed in these three cell groups. (E) The relationship between SA-β-Gal expression and CD4^+^Ki67^−^PD-1^+^T (n = 5 per group). (F) The CD4^+^PD-1^+^T cells and CD4^+^PD-1^−^T cells were purified using immunomagnetic beads and labeled with CFSE, and the cell proliferative capacity was analyzed by flow cytometry after incubating in RPMI-1640 complete medium containing 10 % FBS in a 37 °C for 5 days (n = 3 per group). (G) Relative mRNA expression levels of *P53*, *P21*, and *P16* in purified RA-CD4^+^PD-1^+^T cells and RA-CD4^+^PD-1^−^T cells were analyzed by qPCR (n = 3 per group). (H) The proportion of CD28^neg^, CD27^neg^, CD57^+^ T cells in CD4^+^PD-1^+^T cells and CD4^+^PD-1^−^T cells from RA patients (n = 8) and HC (n = 8) was detected by flow cytometry. Symbols represent individual subjects. ns, no significance; ∗, *P* < 0.05; ∗∗, *P* < 0.01; ∗∗∗, *P* < 0.001.

It has been reported that senescent T cells highly express PD-1 [[14], [15], [16],^30^]. In order to identify PD-1 as a senescence marker, we first assessed for alterations in RA CD4^+^PD-1^+^T cell gene signatures using previously published data (GSE199490) [17]. And conducted KEGG pathway analysis. We found significant enrichment of the cell cycle and the P53 signaling pathway within the CD4^+^PD-1^+^T cells (Fig. 1A). Concomitantly, we showed a significant positive correlation between *PDCD1* (encoding PD-1) and *P16*, *P21,* and SASP-related genes (*IFNG*, *IL-21*, *CXCL13*, *PRF1,* and GZMK) (Fig. 1B). Importantly, our study demonstrated that the proportion of CD4^+^PD-1^+^T cells was significantly correlated with age (Fig. 1C). We divided RA CD3^+^CD4^+^T cells into three subsets: Ki67^−^SA-β-Gal^high^, Ki67^−^SA-β-Gal^mid^, and Ki67^−^SA-β-Gal^low^. Flow cytometric analysis revealed that PD-1 was highly expressed in Ki67^−^SA-β-Gal^high^ cells (Fig. 1D), and the proportion of CD4^+^PD-1^+^Ki67^−^T cells was significantly positively correlated with SA-β-Gal activity (Fig. 1E). In addition, we found that CD4^+^PD-1^+^T cells exhibited a reduced proliferative capacity (Fig. 1F), coinciding with the upregulation of gene expression of *P53, P21,* and *P16* (Fig. 1G). Furthermore, we found that compared with CD4^+^PD-1^−^T cells, CD4^+^PD-1^+^T cells displayed reduced CD27 and CD28 expression and elevated CD57 expression [13,31,32] (Fig. 1H). Additionally, we revealed that RA CD4^+^PD-1^+^T cells exhibited an effector memory phenotype (Fig. S2). Collectively, these findings suggest that CD4^+^PD-1^+^T cells exhibit characteristics associated with T cell senescence.

### 3.2 Expanded CD4^+^PD-1^+^T cells accelerate arthritis progression in CIA mice

**Fig. 2:**
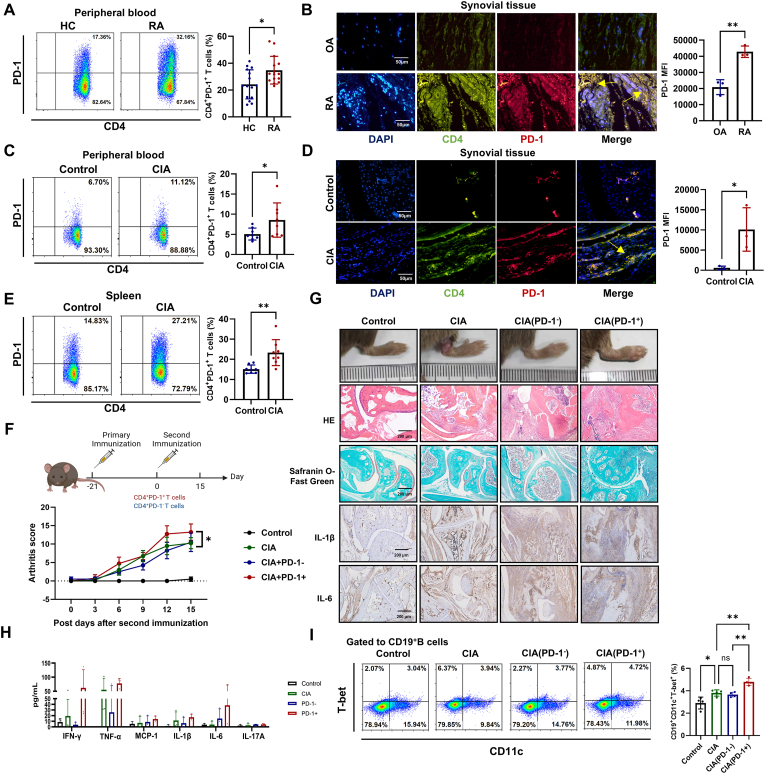
**Expanded CD4^+^PD-1^+^T cells accelerate arthritis progression in CIA mice.** (A) The proportion of CD4^+^PD-1^+^T cells in the peripheral blood of RA patients (n = 15) and HC (n = 15) was detected by flow cytometry. (B) Immunofluorescent staining of CD4^+^PD-1^+^T cells in the synovium of RA (n = 3) and OA patients (n = 3). (C–E) The CD4^+^PD-1^+^T cells in CIA and control mice peripheral blood (n = 8 per group), synovium (n = 3 per group), and spleen (n = 8 per group) were compared. (F) CD4^+^PD-1^+^T cells and CD4^+^PD-1^−^T cells were isolated from the spleens of CIA mice. And 1 × 10^6^ CD4^+^PD-1^+^T cells and CD4^+^PD-1^−^T cells were respectively transferred into CIA mice via tail vein injection on 21 days after immunization (n = 4 per group). (G) The degree of arthritis was observed, and HE staining was used to observe the infiltration of inflammatory cells in the synovium of CIA mice joints. Safranin O-Fast Green staining was used to detect cartilage damage. Immunohistochemical staining was used to detect the levels of IL-1β and IL-6 in the synovium of CIA mice (n = 3 per group). (H) The inflammatory cytokines (IFN-γ, TNF-α, MCP-1, IL-1β, IL-6, IL-17A) levels in the serum of CIA mice (n = 3 per group). (I) The proportion of aging-associated B cells (CD19^+^CD11c^+^T-bet^+^) in the spleen of CIA mice was analyzed by flow cytometry (n = 4 per group). Symbols represent individual subjects. ns, no significance; ∗, *P* < 0.05; ∗∗, *P* < 0.01.

Our study further confirmed the accumulation of CD4^+^PD-1^+^T cells in the peripheral blood and synovium in RA patients, as well as in the peripheral blood, spleen, and synovium of CIA mice (Fig. 2A–E and Fig. S3). Subsequently, to elucidate the pathogenic role of CD4^+^PD-1^+^T cells in the development of RA, we performed adoptive transfer experiments in CIA mice using either CD4^+^PD-1^+^T cells or CD4^+^PD-1^−^T cells as the cellular intervention (Fig. 2F). Our findings revealed that compared to the mice receiving CD4^+^PD-1^−^T cells adoptive transfer, CIA mice subjected to adoptive transfer of CD4^+^PD-1^+^T cells increased the severity of arthritis, indicated by a higher arthritis score, substantial enhancement of synovial cell infiltration, pronounced cartilage damage, and enhanced expression of IL-1β and IL-6 in the synovium (Fig. 2G). Additionally, the adoptive transfer of CD4^+^PD-1^+^T cells also led to elevated levels of pro-inflammatory cytokines in CIA mice serum (Fig. 2H). Notably, we observed a significant upregulation of the proportion of age-associated B cells [33] (CD19^+^CD11c^+^T-bet^+^) (Fig. 2I) and Th1 cells (Fig. S4) in the spleen of CIA mice that received adoptive transfer of CD4^+^PD-1^+^T cells. These results underscore the potential of CD4^+^PD-1^+^T cells as key mediators in the inflammatory progression of RA.

### 3.3 RA CD4^+^PD-1^+^T cells are prone to pro-inflammatory phenotype in vitro

**Fig. 3:**
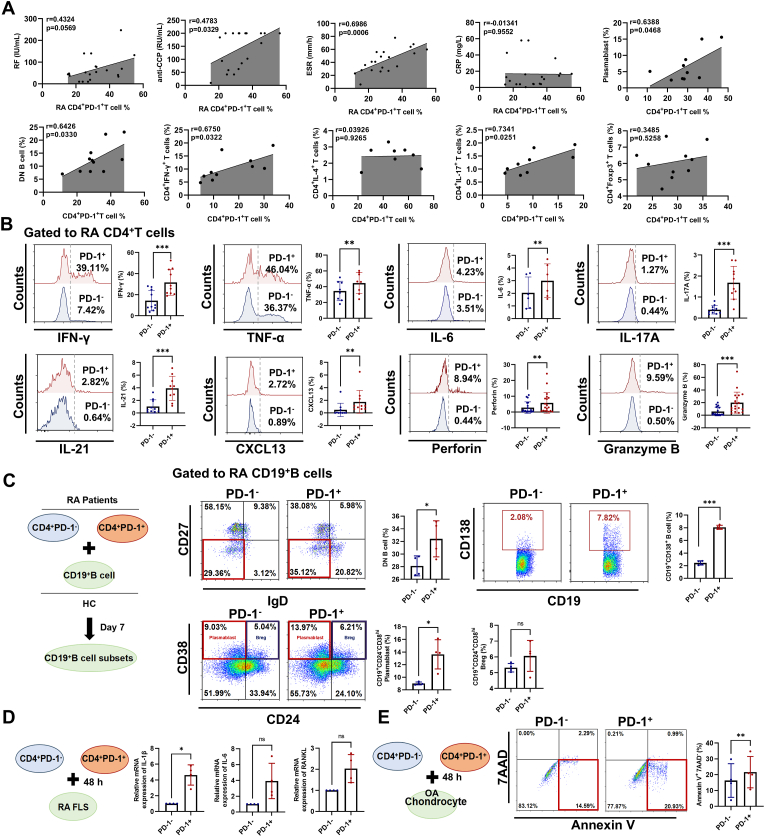
**RA CD4^+^PD-1^+^T cells are prone to pro-inflammatory phenotype in vitro.** (A) The correlation analysis between the CD4^+^PD-1^+^T cells and RA disease activity (RF, *anti*-CCP, CRP, and ESR), plasmablasts, the senescent B-cell subpopulations (DN B cell), Th1 (CD4^+^IFN-γ^+^T), Th2 (CD4^+^IL-4^+^T), Th17 (CD4^+^IL-17^+^T), and Treg (CD4^+^Foxp3^+^T) cells. (B) The expression of cytokines (IFN-γ, n = 10; TNF-α, n = 8; IL-6, n = 6; IL-17A, n = 10; IL-21, n = 10; CXCL13, n = 10) and cytotoxic molecules (Perforin, n = 19; Granzyme B, n = 19) in CD4^+^PD-1^+^T cells and CD4^+^PD-1^−^T cells were analyzed by flow cytometry. (C–E) RA CD4^+^PD-1^+^T cells and CD4^+^PD-1^−^T cells from RA patients were co-cultured with B cells (n = 4 per group), RA fibroblast-like synoviocytes (FLS, n = 4 per group), and OA chondrocytes (n = 4 per group). (C) The DN B cells, plasma cells, plasmablasts, and Breg cells were detected by flow cytometry. (D) The gene expression of *IL-1β*, *IL-6, and RANKL* from RA FLS was detected by qPCR. (E) The apoptosis of OA chondrocytes was detected by flow cytometry. Symbols represent individual subjects. ns, no significance; ∗, *P* < 0.05; ∗∗, *P* < 0.01; ∗∗∗, *P* < 0.001.

To describe the pathological role of CD4^+^PD-1^+^T cells, we first conducted a multiparametric correlation analysis on the frequency of CD4^+^PD-1^+^T cells and serological indicators, as well as T and B cell subsets in RA patients. Our data demonstrated a significant positive correlation between the frequency of CD4^+^PD-1^+^T cells and RA disease activity (*anti*-CCP and ESR), plasmablasts, the senescent B-cell subpopulations (double negative B cells, DN B cells), Th1 cells, and Th17 cells (Fig. 3A). Senescent T cells secrete a range of mediators, including pro-inflammatory cytokines, granzymes, and perforin, which have been recognized as effector T cells in autoimmune tissue inflammation [34]. We observed an enhancement in the secretion of pro-inflammatory cytokines (IFN-γ, TNF-α, IL-6, IL-17A, IL-21, and CXCL13), the cytotoxic molecules (Perforin and Granzyme B) (Fig. 3B) and the expression of relative transcription factors (T-bet, Eomes, BATF, and Blimp1) (Fig. S5) by RA CD4^+^PD-1^+^T cells.

To investigate whether CD4^+^PD-1^+^T cells influenced other cells within RA patients, we conducted cell co-culture experiments and examined their impact on B cell subsets, FLS, and chondrocytes. Our findings indicated that RA CD4^+^PD-1^+^T cells augmented the proportions of DN B cells, plasma cells, and plasmablasts of RA patients in vitro (Fig. 3C). Furthermore, RA CD4^+^PD-1^+^T cells were shown to enhance the secretion of IL-1β by RA-FLS (Fig. 3D) and promote the apoptosis of chondrocytes of OA patients (Fig. 3E). These findings suggest that RA senescent CD4^+^PD-1^+^T cells secrete SASP and cytotoxic molecules, which may thereby influence other effector cells, contributing to the aggravation of RA progression.

### 3.4 RA CD4^+^PD-1^+^T cells exhibit decreased DRP1 expression, which promotes MtROS accumulation

**Fig. 4:**
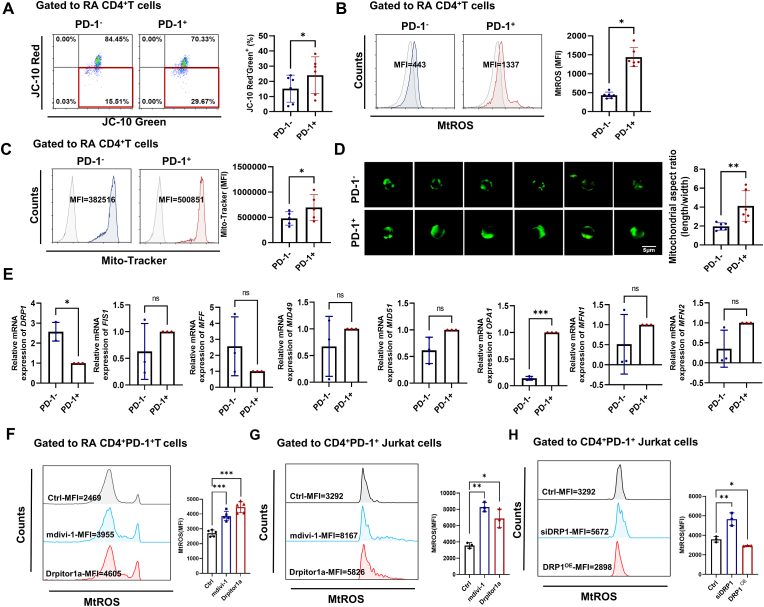
**RA CD4^+^PD-1^+^T cells exhibit decreased DRP1 expression, which promotes MtROS accumulation.** (A) Mitochondrial membrane potential (MMP) in RA CD4^+^PD-1^+^T cells and CD4^+^PD-1^−^T cells was detected using JC-10 staining (n = 6). (B) MtROS levels were assessed using MitoSOX-Red (n = 6). (C–D) Mitochondrial quality (n = 5) and morphology (n = 6) were observed using Mito-Tracker. (E) The relative mRNA expression of mitochondrial dynamics-related molecules was detected by RT-qPCR (n = 3). (F–G) The MtROS levels in RA CD4^+^PD-1^+^T cells (n = 5) and CD4^+^PD-1^+^ Jurkat cells (n = 3) were detected following treatment with two distinct DRP1 inhibitors, mdivi-1 and Drpitor1a. (H) The MtROS levels in CD4^+^PD-1^+^ Jurkat cells were measured by flow cytometry following DRP1 knockdown or overexpression in Jurkat cells using siRNA (n = 3) or plasmid transfection (n = 3). Symbols represent individual subjects. ns, no significance; ∗, *P* < 0.05; ∗∗, *P* < 0.01; ∗∗∗, *P* < 0.001.

Mitochondrial dysfunction is a hallmark of cell senescence [13]. We found that compared to RA CD4^+^PD-1^−^T cells, RA CD4^+^PD-1^+^T cells exhibited a decreased MMP (Fig. 4A), elevated levels of MtROS (Fig. 4B), increased mitochondrial mass (Fig. 4C), and hyperfused and elongated mitochondrial shape (Fig. 4D), which are consistent with the characteristics of mitochondrial morphology in senescent cells [22]. Studies suggest that cell senescence is associated with unbalanced mitochondrial dynamics [23]. Our data showed that the gene expression of fission-associated *DRP1* was downregulated while that of fusion-associated *OPA1* was upregulated in RA CD4^+^PD-1^+^T cells (Fig. 4E). Interestingly, the inhibitor of DRP1 (mdivi-1) could further promote the production of MtROS, whereas the inhibitor of OPA1 (MYLS22) had no significant impact on MtROS in RA CD4^+^PD-1^+^T cells (Fig. S6), which implies that decreased DRP1 is prominent in CD4^+^PD-1^+^T cells dysfunction.

To further validate the inhibitory effect of DRP1 on MtROS production in RA CD4^+^PD-1^+^T cells, we employed two distinct DRP1 inhibitors (mdivi-1 and Drpitor1a). We found that pharmacological inhibition of DRP1 induced MtROS accumulation in RA CD4^+^PD-1^+^T cells and CD4^+^PD-1^+^ Jurkat cells (Fig. 4F and G). Besides, we genetically modulated DRP1 in Jurkat cells through siRNA-mediated knockdown and plasmid-driven overexpression. The efficiency of DRP1 overexpression and knockdown was assessed by Western blot analysis, as shown in Fig. S7A and S7B. Our results revealed that DRP1 depletion significantly elevated MtROS levels, whereas DRP1 overexpression reduced MtROS production in CD4^+^PD-1^+^ Jurkat cells (Fig. 4H).

### 3.5 Scavenging of MtROS can attenuate SASP production of RA CD4^+^PD-1^+^T cells, contributing to decreased joint inflammation in CIA mice

**Fig. 5:**
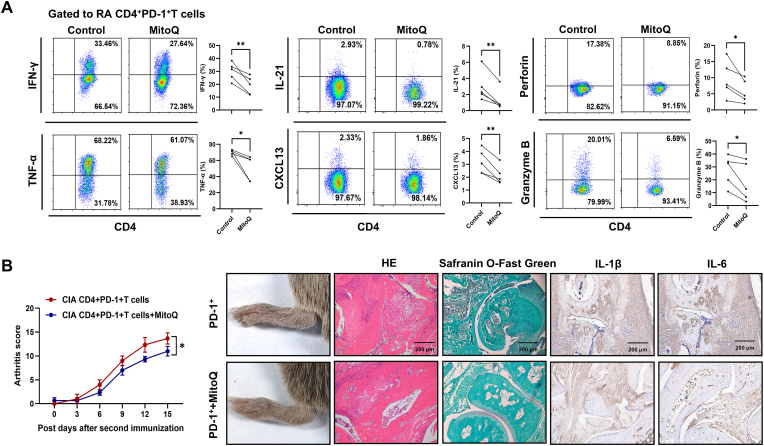
**Scavenging of MtROS can attenuate SASP production of RA CD4^+^PD-1^+^T cells, contributing to decreased joint inflammation in CIA mice.** (A) CD4^+^T cells from RA patients were treated with mitoquinone (n = 5 per group), and the levels of cytokines (IFN-γ, TNF-α, IL-21, and CXCL13) and cytotoxic molecules (Perforin, Granzyme B) in CD4^+^PD-1^+^T cells were analyzed by flow cytometry. (B) CD4^+^PD-1^+^T cells were sorted from the spleens of CIA mice, treated with or without mitoquinone (200 nM for 4 h), and then adoptively transferred into other CIA mice (n = 3 per group). The degree of arthritis was observed, and HE staining was used to assess synovial inflammatory cell infiltration in the CIA mice. Safranin O-Fast Green staining was used to detect cartilage damage. Immunohistochemical staining was used to detect the expression of IL-1β and IL-6 in the synovium of CIA mice. Symbols represent individual subjects. ∗, *P* < 0.05; ∗∗, *P* < 0.01.

As mentioned, decreased DRP1 in CD4^+^PD-1^+^T cells showed increased MtROS production, which plays a pivotal role in the induction of SASP expression [35,36]. We observed in vitro experiment that MtROS scavenging significantly downregulated the secretion of inflammatory cytokines IFN-γ and TNF-α, the cytotoxic molecules Perforin and Granzyme B (Fig. 5A), and transcription factors (T-bet, Eomes, BATF, and Blimp1) (Fig. S8) in RA CD4^+^PD-1^+^T cells. To further investigate the effects of MtROS on the pathogenic roles of CD4^+^PD-1^+^T cells, we pretreated CD4^+^PD-1^+^T cells derived from CIA mice spleen with MitoQ to scavenge MtROS, and then adoptively transferred either the MitoQ-treated or untreated CD4^+^PD-1^+^T cells into CIA mice. The results showed that CIA mice receiving the adoptive transfer of MitoQ-treated CD4^+^PD-1^+^T cells exhibited lower joint inflammation scores, less severe joint swelling, and reduced inflammatory cell infiltration in the synovium compared to mice receiving untreated CD4^+^PD-1^+^T cells (Fig. 5B). These results suggest that scavenging MtROS can attenuate the pathogenic effects of CD4^+^PD-1^+^T cells, contributing to decreased CIA joint inflammation.

### 3.6 Decreased DRP1 inhibits mitophagy in RA CD4^+^PD-1^+^T cells, leading to MtROS accumulation and subsequent SASP production

**Fig. 6:**
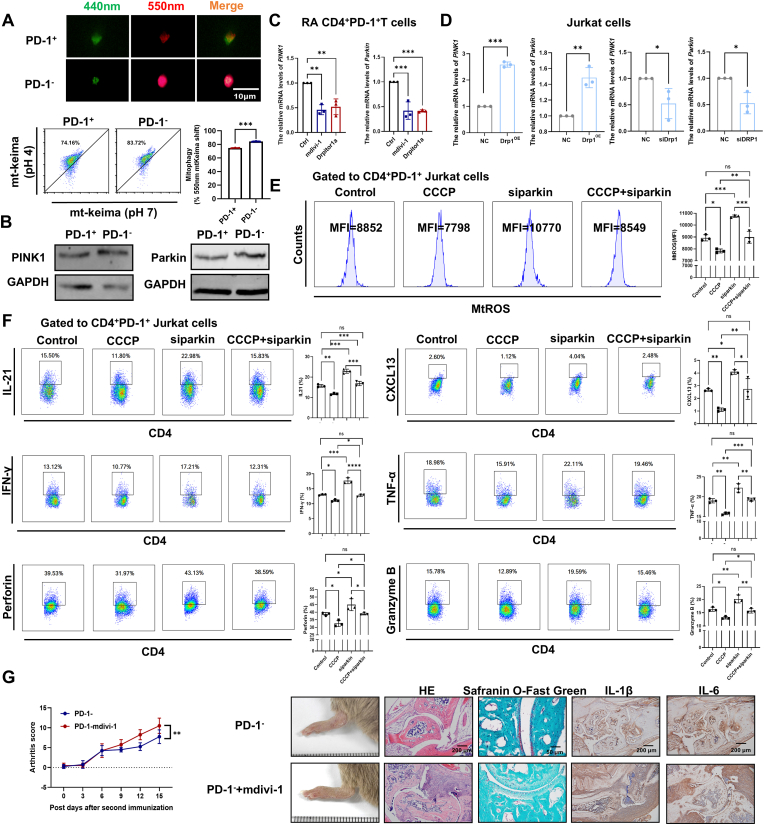
**Decreased DRP1 inhibits mitophagy in RA CD4^+^PD-1^+^T cells, leading to MtROS accumulation and subsequent SASP production.** (A) Mitophagy activity was assessed using fluorescence microscopy and flow cytometry with the mt-Keima reporter (n = 3 per group). Enhanced mitophagy was indicated by increased fluorescence under 550 nm excitation (red) and decreased fluorescence under 440 nm excitation (green). For flow cytometry, the proportion of red-positive cells (mt-Keima at pH 4, 550 nm excitation) was quantified. (B) The levels of mitophagy proteins (PINK1 and Parkin) in RA CD4^+^PD-1^+^T cells and CD4^+^PD-1^−^T cells were detected by Western blot (n = 3). (C) RA CD4^+^PD-1^+^T cells were treated with DRP1 inhibitors (mdivi-1 and Drpitor1a), then the gene expression of *PINK1* and *Parkin* was detected by qPCR (n = 3 per group). (D)The gene expression of *PINK1* and *Parkin* was detected by qPCR following DRP1 knockdown or overexpression in Jurkat cells using siRNA (n = 3 per group) or plasmid transfection (n = 3 per group). (E–F) The levels of MtROS, SASP components (IL-21, CXCL13, IFN-γ, TNF-α), and cytotoxic molecules (Perforin, and Granzyme B) in CD4^+^PD-1^+^ Jurkat cells were analyzed under three experimental regimens: treatment with CCCP, siParkin transfection, and sequential siParkin transfection followed by CCCP exposure (n = 3 per group). (G) CD4^+^PD-1^−^T cells were sorted from the spleens of CIA mice, treated with or without mdivi-1 (4 h), and then adoptively transferred into other CIA mice (n = 4 per group). The degree of arthritis was observed, and HE staining was used to observe the infiltration of inflammatory cells in the synovium of CIA mice joints. Safranin O-Fast Green staining was used to detect cartilage damage. Immunohistochemical staining was used to detect the expression of IL-1β and IL-6 in the synovium of CIA mice. Symbols represent individual subjects. ns, no significance; ∗, *P* < 0.05; ∗∗, *P* < 0.01; ∗∗∗, *P* < 0.001.

As DRP1 regulates mitophagy [37,38], we detected the levels of mitophagy and mitophagy-related proteins (PINK1 and Parkin) in RA CD4^+^PD-1^+^T cells and CD4^+^PD-1^-^ T cells. We found that mitophagy was reduced in RA CD4^+^PD-1^+^T cells (Fig. 6A), concomitant with reduced expression of PINK1 and Parkin (Fig. 6B). The inhibitors of DRP1 could downregulate the mRNA expressions of *PINK1* and *Parkin* in RA CD4^+^PD-1^+^T cells (Fig. 6C). Furthermore, genetic modulation of *DRP1* in Jurkat cells demonstrated its regulatory capacity in mitophagy-associated gene expression, with *DRP1* overexpression upregulating the mRNA expressions of both *PINK1* and *Parkin*, whereas *DRP1* knockdown suppressed their expressions (Fig. 6D).

It has been reported that impaired mitophagy promotes mtROS production [38]. While CCCP is widely regarded as a pharmacological inducer of mitophagy [39,40] and Parkin is a recognized mitophagy marker [41,42]. We assessed mitophagy induction by treating cells with CCCP, either with or without Parkin knockdown via siRNA, and analyze Parkin protein levels by using Western blot. As shown in Fig. S7C–S7D, siParkin significantly downregulated Parkin expression, while subsequent CCCP treatment markedly increased Parkin levels. Further, we found that CCCP reduced MtROS levels in RA CD4^+^PD-1^+^T cells (Fig. S9) and CD4^+^PD-1^+^ Jurkat cells (Fig. 6E). Moreover, the knockdown of Parkin exacerbated MtROS accumulation, and the addition of CCCP could partially reduce MtROS production (Fig. 6E). Furthermore, enhanced mitophagy by CCCP significantly attenuated the production of SASP-related cytokines (IL-21, CXCL13, IFN-γ, and TNF-α) and cytotoxic molecules (Perforin and Granzyme B) in RA CD4^+^PD-1^+^T cells (Fig. S9) and CD4^+^PD-1^+^ Jurkat cells (Fig. 6F). Conversely, Parkin interference amplified these inflammatory and cytotoxic signatures in CD4^+^PD-1^+^ Jurkat cells (Fig. 6F). We also observed that the levels of SASP in the CCCP + siParkin group decreased compared with the siParkin group (Fig. 6F). Our results suggest that mitophagy mediated by Parkin may partly be responsible for preventing CD4^+^PD-1^+^T cell senescence.

To further ascertain the pathogenic roles of decreased DRP1 of RA CD4^+^PD-1^+^T cells in CIA mice, we treated CIA mice spleen CD4^+^PD-1^+^ and CD4^+^PD-1^−^T cells with DRP1 inhibitor prior to adoptive transfer into recipient CIA mice. The results demonstrated that compared with CIA mice receiving adoptive transfer of CD4^+^PD-1^−^T cells, those receiving DRP1 inhibitor-treated CD4^+^PD-1^−^T cells exhibited significantly exacerbated joint inflammation (Fig. 6G). However, no difference in joint inflammation severity was observed in CIA mice between receiving CD4^+^PD-1^+^ T cells and DRP1 inhibitor-treated CD4^+^PD-1^+^T cells, which may be attributable to the already significantly upregulated inflammatory responses present in both groups (Fig. S10).

### 3.7 PD-1 signaling inhibits the DRP1 expression via suppressing HIF-1α and promotes cellular senescence in RA CD4^+^PD-1^+^T cells

**Fig. 7:**
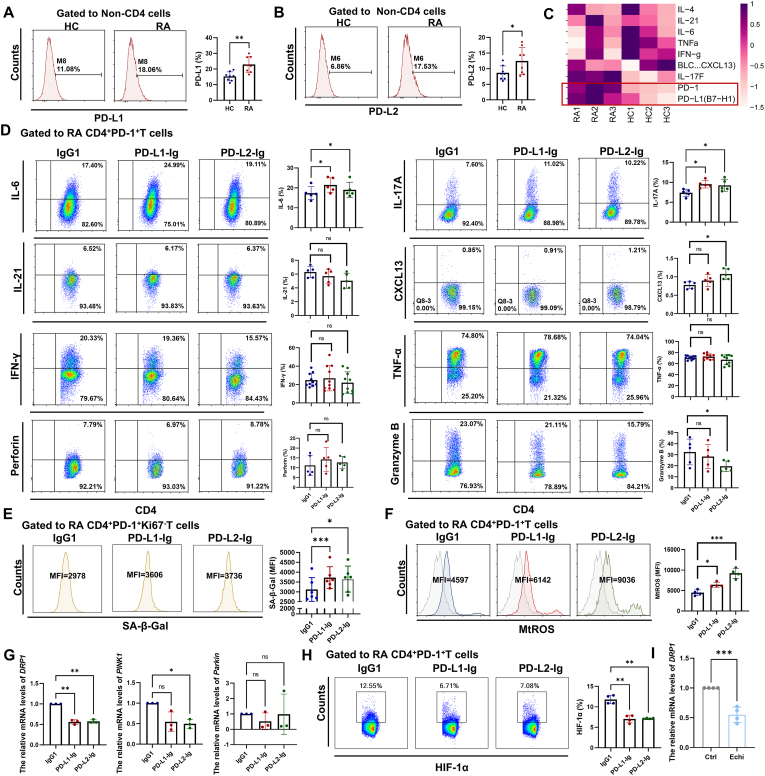
**PD-1 signaling inhibits the DRP1 expression via suppressing HIF-1α and promotes cellular senescence in RA CD4^+^PD-1^+^T cells.** (A–B) The expression of PD-L1 and PD-L2 in non-CD4^+^T cells in RA patients (n = 8) and HC (n = 8) was detected by flow cytometry. (C) Heat map of patient-group-supervised clustering revealed the landscape of protein expression among the serum from RA patients and HC with protein array analysis (n = 3 per group). (D–H) CD4^+^T cells from RA patients were sorted and stimulated with IgG1 (10 μg/mL), PD-L1-Ig (10 μg/mL), or PD-L2-Ig (10 μg/mL) for 24–72 h. (D) The levels of cytokines (IL-6, n = 5; IL-17A, n = 5; IL-21, n = 5; CXCL13, n = 5; IFN-γ, n = 10; TNF-α, n = 10), and cytotoxic molecules (Perforin, n = 5; Granzyme B, n = 5) in RA CD4^+^PD-1^+^T cells were analyzed (72 h) by flow cytometry. (E) The levels of SA-β-Gal (48h, n = 6) in RA CD4^+^Ki67^−^PD-1^+^T were detected by flow cytometry. (F–H) The levels of MtROS (24 h, n = 4) in RA CD4^+^PD-1^+^T cells, the gene expression of *DRP1* (24 h, n = 3), *PINK1* (24 h, n = 3), and *Parkin* (24 h, n = 3), and the level of HIF-1α were detected by flow cytometry (72 h, n = 4). *DRP1* mRNA levels in RA CD4^+^PD-1^+^T cells were quantified by qPCR following treatment with HIF-1α inhibitor (Echinomycin, Echi) for 24 h (n = 4). Symbols represent individual subjects. ns, no significance; ∗, *P* < 0.05; ∗∗, *P* < 0.01; ∗∗∗, *P* < 0.001.

Finally, we focused on the potential impact of PD-1 signaling on DRP1 and cellular senescence in RA CD4^+^PD-1^+^T cells. We noted upregulation of PD-1 ligands in non-CD4^+^T cells and the serum of RA patients (Fig. 7A–C). PD-1 signaling is conventionally recognized to suppress IFN-γ, TNF-α, Perforin, and Granzyme B in tumor-infiltrating T cells [43]. However, we found that PD-1 signaling had no significant inhibitory effect on the above cytokines in RA CD4^+^PD-1^+^T cells, but instead potentiated the upregulation of pathogenic inflammatory mediators, including IL-6, IL-17A, and CXCL13 (Fig. 7D). Importantly, upon scavenging MtROS, PD-1 signaling was able to reduce the secretion of IFN-γ, perforin, and granzyme B in RA CD4^+^PD-1^+^T cells (Fig. S11). In addition, we also found that PD-1 signaling could upregulate the SA-β-Gal activity (Fig. 7E), inhibit CD4^+^PD-1^+^T cell proliferation, and upregulate the expression of P53, P16, and P21 (Fig. S12). We further evaluated the effect of PD-1 signaling on mitochondrial function. We found that PD-1 signaling promoted MtROS production (Fig. 7F) and inhibited mRNA expression of *DRP1* and *PINK1* in RA CD4^+^PD-1^+^T cells (Fig. 7G).

Hypoxia inducible factor 1 alpha subunit (HIF-1α) activation, resulting in Drp1_Ser616_ phosphorylation, is involved in mitochondrial fission [44,45]. We further found that PD-1 signaling could inhibit the expression of HIF-1α (Fig. 7H). Treatment with the HIF-1α inhibitor Echinomycin (Echi) could suppress the mRNA expression of *DRP1* (Fig. 7I), indicating that PD-1 signaling may downregulate DRP1 expression by inhibiting HIF-1α.

## 4 Discussion

In this study, we identified CD4^+^PD-1^+^T cells as a key senescent subset in RA. We demonstrated that DRP1 downregulation impaired mitophagy, resulting in MtROS accumulation, which drives the secretion of SASP and cytotoxic factors in these cells. Crucially, we uncovered that PD-1 signaling exacerbates T cell senescence by suppressing DRP1 expression via HIF-1α inhibition (Fig. S13).

Features of accelerated immunosenescence can be identified in adults with chronic inflammatory conditions, such as RA, and are predictive of poor clinical outcomes [46]. Evidence suggests that T cells are most sensitive to senescence, and senescent CD4^+^T cells play a crucial role in accelerating autoimmune diseases and other immune-related pathologies [8]. Identifying and targeting the specific pathogenic senescent T cell subsets may be an effective therapeutic strategy for RA. Our study confirmed that RA CD4^+^T cells undergo senescence, and especially CD4^+^PD-1^+^T cells displayed senescence characteristics. In addition, expanded senescent CD4^+^PD-1^+^T cells accelerated arthritis progression in CIA mice, indicating the key roles of these cells in the pathogenesis of RA. Supporting this, Zhao Peng et al. demonstrated that depleting PD-1-expressing cells ameliorates autoimmune disease [47]. Collectively, these findings highlight the pathogenic role of senescent CD4^+^PD-1^+^T cells in RA and suggest that their depletion may be a promising therapeutic approach.

Senescent T cells develop a characteristic pathogenic SASP, which encompasses growth factors, chemokines, and pro-inflammatory cytokines, acting on adjacent cells [48,49]. In RA microenvironment, multi-cells communication and interaction by cytokines result in a variety of pathological reactions. In our study, we observed that RA CD4^+^PD-1^+^T cells were related to RA disease activity and secreted higher levels of pro-inflammatory cytokines, which have roles in other targets. Indeed, we also found that CD4^+^PD-1^+^T cells augmented the proportions of DN B cells, promoted the secretion of IL-1β by RA-FLS, and OA chondrocyte apoptosis. DN B cells have been reported to expand in RA patients and mediate joint inflammation by secreting inflammatory factors [50]. RA-FLS display an aggressive phenotype that contributes to synovial inflammation [51]. Chondrocyte apoptosis is dedicated to cartilage damage, a key feature of RA patients.

Mitochondrial dysfunction is a closely interconnected hallmark of T cell senescence characterized by increased mitochondrial mass, low MMP, and increased production of MtROS [21,52]. Changes in mitochondrial morphology through fission and fusion processes, known as mitochondrial dynamics, are crucial to maintaining mitochondrial number, size, shape, and distribution [53]. Mitochondrial fusion is mediated by the fusion proteins mitofusin (MFN) 1, MFN2, and optic atrophy 1 (OPA1). Mitochondrial fission is mediated by DRP1, which interacts with four mitochondrial receptor proteins: fission 1 (FIS1), mitochondrial fission factor (MFF), mitochondrial dynamics protein of 49 kDa (MID49), and MID51 [54]. Perturbations in mitochondrial dynamics have been associated with cellular senescence [23]. In our study, we found that RA CD4^+^PD-1^+^T cells exhibited hyperfused and elongated mitochondrial shape. Current methods for quantifying mitochondrial shape via aspect ratio (length/width) may be limited by image quality and technical constraints. However, the combined data, including increased mitochondrial mass and altered expression of mitochondrial dynamics proteins (downregulated DRP1 and upregulated OPA1), supported the conclusion of unbalanced mitochondrial dynamics in CD4^+^PD-1^+^T cells, which are consistent with the characteristics of mitochondrial morphology in senescent cells. Moreover, DRP1 is essential for segregating damaged mitochondrial matrices to maintain normal mitochondrial function [55]. Recent works have shown that inhibition of DRP1 promotes cell senescence [38,56]. In our study, RA CD4^+^PD-1^+^T cells exhibited downregulated expression of the DRP1*,* contributing to the production of MtROS and SASP. However, it has been reported that elevated DRP1 leads to cellular senescence [57,58]. This may be related to the pathogenesis of different diseases.

DRP1-mediated mitochondrial fission separates injured mitochondria, and this is followed by mitophagy [37,38]. Mitophagy acts as a defense mechanism against senescence, aging, and age-related phenotypes, including inflammation [59,60]. Among different mitophagy pathways, PINK1/Parkin is the most extensively studied stress-induced mitophagy pathway [61]. Our results showed that lower DRP1 downregulated the expression of PINK1/Parkin in RA CD4^+^PD-1^+^T cells, and the knockdown of Parkin to prevent mitophagy increased MtROS and SASP production. These findings establish a functional DRP1-mitophagy-SASP axis in RA CD4^+^PD-1^+^T cells, linking mitochondrial dysfunction to pro-inflammatory senescence.

Interaction with either of the ligands, programmed death ligand 1 or 2 (PD-L1 or PD-L2, respectively), PD-1 triggers an inhibitory signal in PD-1-expressing T cells, potentially leading to T cell anergy or exhaustion [17,62]. However, A study reported that PD-1 signaling can promote T cell secretion of cytokines associated with helper B cells [63,64]. In our study, we observed a slight increase in the expression of IL-17A, IL-6, and CXCL13 following PD-1 signaling activation, and no inhibitory effect on IFN-γ and TNF-α, which are downregulated in tumor T cells [65,66]. These results suggest that the PD-1 pathway may exert distinct effects in RA. Additionally, we observed that PD-1 signaling reduced cell proliferation and increased the expression of senescence-associated markers, confirming the known role of PD-1 signaling in the promotion of renal podocyte aging [67]. PD‐1 signaling can prevent DRP1 activation, mitophagy, and mitochondrial fragmentation in both murine and human T cells [68]. Our study also identified that PD-1 signaling inhibited the expression of DRP1, impaired mitophagy, and promoted MtROS production.

HIF-1α is a hypoxia-inducing factor that plays a key role in the process of glycolysis [69]. Recent studies have shown that HIF-1α activation resulting in Drp1_Ser616_ phosphorylation is involved in mitochondrial fission and mitophagy [44,45]. And blocking the PD-1 signal can promote the HIF-1α signaling pathway in tumor cells [70]. Therefore, we hypothesized and verified that the PD-1 signal reduced DRP1 expression by inhibiting HIF-1α in RA CD4^+^PD-1^+^T cells. However, additional experimental studies are required to elucidate the underlying mechanism.

In summary, our findings contribute more substantially to elucidating the link between T cell senescence and RA pathogenesis. Nevertheless, our study has several limitations. First, due to the low transfection efficiency of primary RA CD4^+^T cells, we performed gene overexpression and knockout experiments in Jurkat cell lines instead. Second, while the CIA model is typically established in DBA/1J mice due to their high susceptibility, which is less amenable to genetic manipulation, alternative RA mouse models are necessary to further explore how DRP1 downregulation influences CD4^+^PD-1^+^T cell senescence. Finally, the mechanism by which the PD-1 signaling pathway suppresses DRP1 through HIF-1α requires additional experimental validation, both in vitro and in vivo.

## CRediT authorship contribution statement

**Ziran Bai:** Writing – original draft, Methodology. **Jinyi Ren:** Writing – original draft, Methodology. **Jiaqing Liu:** Methodology. **Cheng Zhang:** Software, Methodology. **Huina Huang:** Writing – original draft. **Xiangge Zhao:** Writing – original draft. **Xianmei Chen:** Methodology. **Jing Wei:** Writing – original draft. **Jingjing Qi:** Writing – original draft. **Siwen Yang:** Writing – original draft. **Weiping Li:** Writing – original draft. **Yawei Tang:** Writing – review & editing. **Guan Wang:** Writing – review & editing. **Xia Li:** Writing – review & editing, Methodology, Funding acquisition.

## Ethics approval

This study was approved by the Ethics Committee of the Second Hospital of Dalian Medical University (No. 2023-253). Participants gave informed consent to participate in the study before taking part. The animal experiment was approved by the Laboratory Animal Ethics Committee of Dalian Medical University (AEE24013).

## Data availability statement

Data are available from the corresponding author upon reasonable request.

## Funding

National Natural Science Foundation of China (82071834, 82101896, 82271839, and 82302047), Liaoning Provincial Education Department Basic Research Project (LJ212410161057 and LJ212410161034), Dalian Medical University Interdisciplinary Research Cooperation Project Team Funding (JCHZ2023010).

## Declaration of competing interest

The authors declare that they have no known competing financial interests or personal relationships that could have appeared to influence the work reported in this paper.

## Data Availability

Data will be made available on request.
