# Supplementary material for: DRP1 downregulation impairs mitophagy, driving mitochondrial ROS and SASP production in rheumatoid arthritis CD4+PD-1+T cells
Source: Redox Biol. 2025 Aug 9;86:103818. doi: 10.1016/j.redox.2025.103818 (PMC12390946; doi:10.1016/j.redox.2025.103818)
Supplement: Multimedia component 1 [file mmc1.docx]

Supplementary Material

## Supplementary Tables

Supplemental Table 1. Clinical and laboratory characteristics of RA patients for this study.

|  | RA | HC |
| --- | --- | --- |
| Age (years) | 62.0 (49-72) | 58.0 (49-72) |
| Male/female | 23/72 | 14/36 |
| Disease duration (years) | 4.0 (3.0-7.0) | - |
| Current NSAID users | 41 (43.1%) | - |
| Current DMARD users | 56 (58.9%) | - |
| Current TCM users | 18 (18.9%) | - |
| No systemic therapy patients | 13 (13.7%) | - |
| DAS28 | 4.50 (3.25-5.32) | - |
| ESR(mm/h) | 27.00 (11.00-54.00) | - |
| CRP(mg/L) | 13.50 (3.69-42.7) | - |
| RF(IU/mL) | 105.00 (43.4-309.00) | - |
| Anti-CCP(U/mL) | 120.43 (27.49-184.21) | - |

DAS28: Disease Activity Score 28; ESR: Erythrocyte sedimentation rate; CRP: C-reactive protein; RF: Rheumatoid factor; anti-CCP: anti-cyclic peptide containing citrulline.

##### Supplemental Table 2. Antibodies used for Flow cytometry

| Antibodies | Source |
| --- | --- |
| Anti-Human CD4 FITC | Biolegend |
| Anti-Human CD4 APC | Biolegend |
| Anti-Human CD4 APC-Cyanine7 | Biolegend |
| Anti-Human PD-1 PE-Cyanine7 | Biolegend |
| Anti-Human TNF-α APC | Biolegend |
| Anti-Human CD57 APC-Cyanine7 | Biolegend |
| Anti-Human CCR7 APC | Biolegend |
| Anti-Human CD45RO APC-Cyanine7 | Biolegend |
| Anti-Human perforin FITC | Biolegend |
| Anti-Human granzyme B PE | Biolegend |
| Anti-Human CXCL13 APC | Biolegend |
| Anti-Human CD28 PE | Biolegend |
| Anti-Human IgD PE | eBioscience |
| Anti-Human CD27 Percpcy5.5 | eBioscience |
| Anti-Human ICOS PE | eBioscience |
| Anti-Human CXCR5 APC | eBioscience |
| Anti-Human IL-17A PE | eBioscience |
| Anti-Human IFN-γ APC | eBioscience |
| Anti-Human IL-4 PE | eBioscience |
| Anti-Human CD138 PE | eBioscience |
| Anti-Human CD38 FITC | eBioscience |
| Anti-Human PD-1 FITC | Biolegend |
| Anti-Human CD4 Percpcy5.5 | Biolegend |
| Anti-Human CD69 PE | Biolegend |
| Anti-Human IL-6 APC | Biolegend |
| Anti T-bet FITC | Biolegend |
| Anti-Human Eomes FITC | Biolegend |
| Anti-Human CD4 APC | Biolegend |
| Anti-Human CD19 APC | Biolegend |
| Anti-Human IL-21 APC | Biolegend |
| Anti-Human IL-10 APC | Biolegend |
| Anti-Mouse CD4 FITC | Biolegend |
| Anti-Mouse PD-1 APC-Cyanine7 | Biolegend |
| Anti-Mouse IFN-γ APC | Biolegend |
| Anti-Mouse IL-4 APC | Biolegend |
| Anti-Mouse IL-17A APC | Biolegend |
| Anti-Mouse Foxp3 PE | Biolegend |
| Anti-Mouse CD11c PE-Cyanine7 | Biolegend |
| Anti-Mouse CD138 APC | Biolegend |
| Anti-Mouse CD19 APC-Cyanine7 | Biolegend |

##### Supplemental Table 3. Primers used for quantitative PCR

| Genes | Primers |
| --- | --- |
| Human-*β-actin*-Forward | 5’-CATGTACGTTGCTATCCAGGC-3’ |
| Human-*β-actin*-Reverse | 5’-CTCCTTAATGTCACGCACGAT-3’ |
| Human-*P53*-Forward | 5’-TTGCAATAGGTGTGCGTCAGA-3’ |
| Human-*P53*-Reverse | 5’-AGTGCAGGCCAACTTGTTCAG-3’ |
| Human-*P21*-Forward | 5’-TGGACCTGTCACTGTCTTGT-3’ |
| Human-*P21*-Reverse | 5’-TCCTGTGGGCGGATTA-3’ |
| Human-*P16*-Forward | 5’-CCACCCCGCTTTCGTAGTT-3’ |
| Human-*P16*-Reverse | 5’-CCACATGAATGTGCGCTTAGG-3’ |
| Human-*IL-6*-Forward | 5’-CCTTCGGTCCAGTTGCCTTCTC-3’ |
| Human-*IL-6*-Reverse | 5’-CCAGTGCCTCTTTGCTGCTTTC-3’ |
| Human-*IL-1β*-Forward | 5’-GCTGAGGAAGATGCTGGTTC-3’ |
| Human-*IL-1β*-Reverse | 5’-GTGATCGTACAGGTGCATCG-3’ |
| Human-*OPA1*-Forward | 5’-TAGTTCTCGGGAGTTTG-3’ |
| Human-*OPA1*-Reverse | 5’-TATGGTCTCAGGGCTAA-3’ |
| Human-*MFN1*-Forward | 5’-TACCACTCCAGCAACGC-3’ |
| Human-*MFN1*-Reverse | 5’-CAATAATGATGCCCATAGAA-3’ |
| Human-*MFN2*-Forward | 5’-CAAGACTATAAGCTGCGAATT-3’ |
| Human-*MFN2*-Reverse | 5’-GAGGACTACTGGAGAAGGGT-3’ |
| Human-*DRP1*-Forward | 5’-AGGTGCCTGTAGGTGAT-3’ |
| Human-*DRP1*-Reverse | 5’-TGATGTTGCCATATCTGTA-3’ |
| Human-*Mid49*-Forward | 5’-AAGCGGTTCATTGACAGGG-3’ |
| Human-*Mid49*-Reverse | 5’-CTAAGGGCAGCAGGTGGAG-3’ |
| Human-*Mid51*-Forward | 5’-CCATTCGGGGAAAAGGAG-3’ |
| Human-*Mid51*-Reverse | 5’-GAGTCTGTGGGAAGGGTCTG-3’ |
| Human-*FIS1*-Forward | 5’-CTGTGGAGGACCTGCTGA-3’ |
| Human-*FIS1*-Reverse | 5’-ACGATGCCTTTACGGATG-3’ |
| Human-*MFF*-Forward | 5’-GCTGACCTGGAGCAAGA-3’ |
| Human-*MFF*-Reverse | 5’-AAGGGAGTGGACTGGATAA-3’ |
| Human-*PINK1*-Forward | 5’-GCCTCATCGAGGAAAAACAGG-3’ |
| Human-*PINK1*-Reverse | 5’-GTCTCGTGTCCAACGGGTC-3’ |
| Human-*Parkin*-Forward | 5’-GTGTTTGTCAGGTTCAACTCCA-3’ |
| Human-*Parkin*-Reverse | 5’-GAAAATCACACGCAACTGGTC-3’ |

## Supplementary Figures


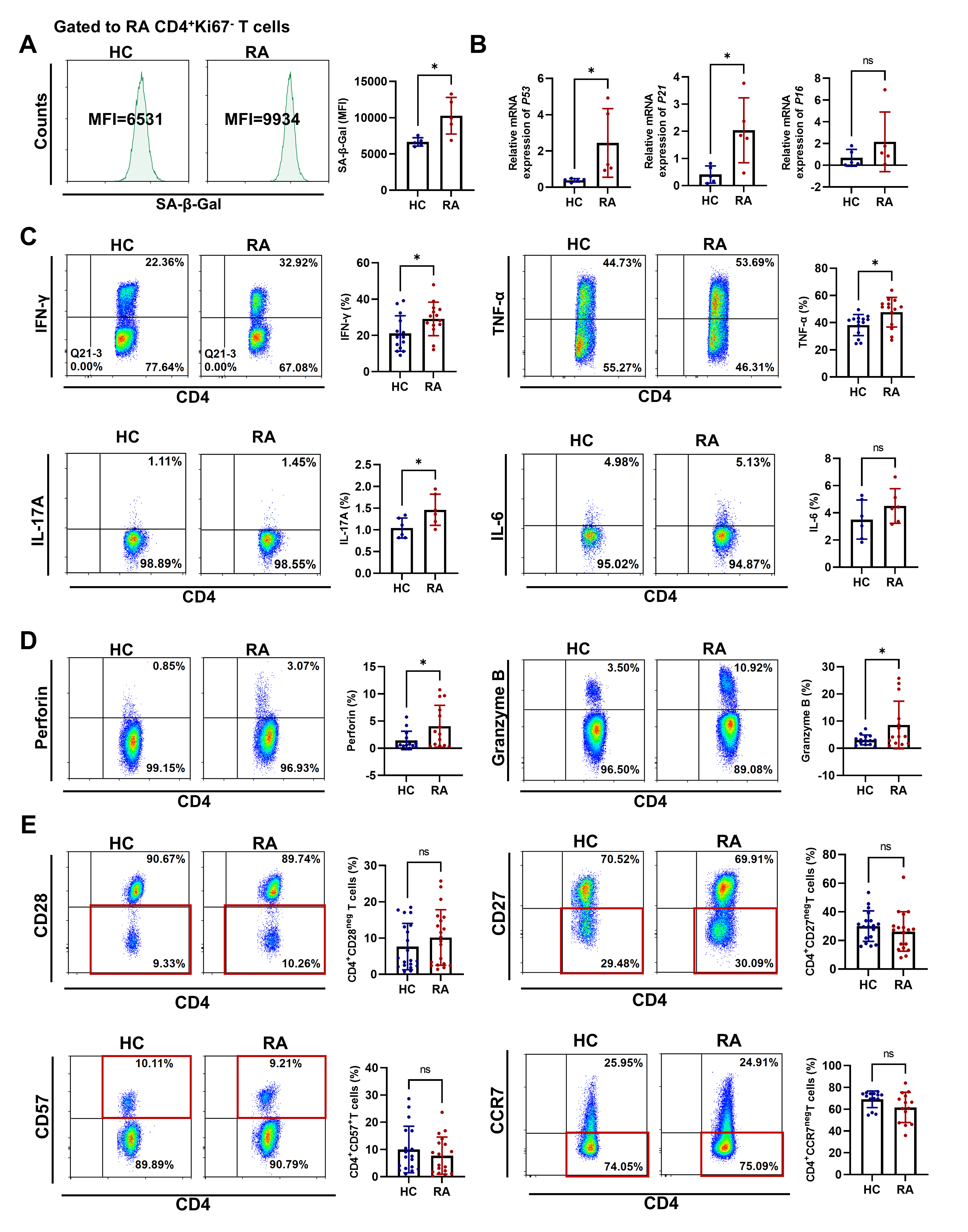


**Figure S1. CD4^+^T cell senescence in RA patients.** (A) CD4^+^T cells were isolated from RA patients and healthy controls (HC), and the SA-β-Gal expression in CD4^+^Ki67^-^T cells was determined by flow cytometry (n=5). (B) Relative mRNA expression levels of *P53*, *P21*, and *P16* in CD4^+^T cells from RA patients and HC were analyzed by qPCR (n=5). (C-E) The expression of cytokines (IFN-γ, n=14; TNF-α, n=14; IL-17A, n=6; IL-6, n=6), cytotoxic molecules (Perforin, n=14; Granzyme B, n=14), and the proportion of CD4^+^CD28^neg^ (n=20), CD4^+^CD27^neg^ (n=18), CD4^+^CD57^+^ (n=18), and CD4^+^CCR7^neg^ (n=14) in RA patients and HC were analyzed by flow cytometry. Symbols represent individual subjects. ns, no significance; *, *P* < 0.05.


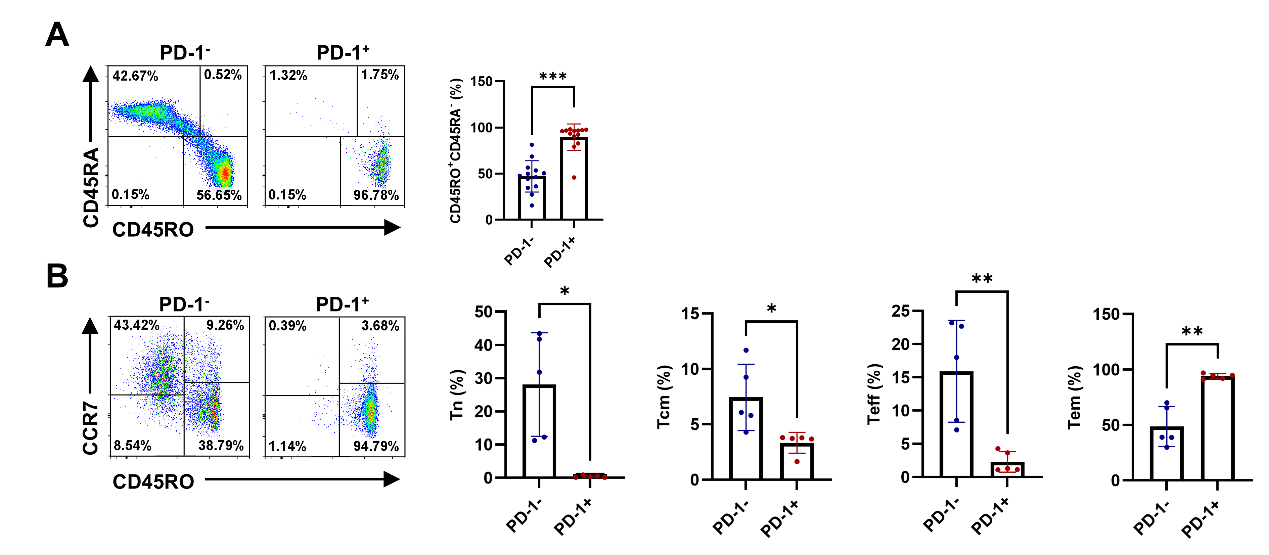


**Figure S2. RA CD4^+^PD-1^+^T cells exhibit an effector memory phenotype.** (A) The proportion of CD45RO^+^CD45RA^-^T cells in CD4^+^PD-1^+^T cells and CD4^+^PD-1^-^T cells from RA patients was determined by flow cytometry (n=13). (B) The frequencies of RA naïve T cells (Tn), central memory T cells (Tcm), effector T cells (Teff), and effector memory T cells (Tem) in RA CD4^+^PD-1^+^T cells and RA CD4^+^PD-1^-^T cells were shown (n=5). Symbols represent individual subjects. *, *P* < 0.05; **, *P* < 0.01; ***, *P* < 0.001.


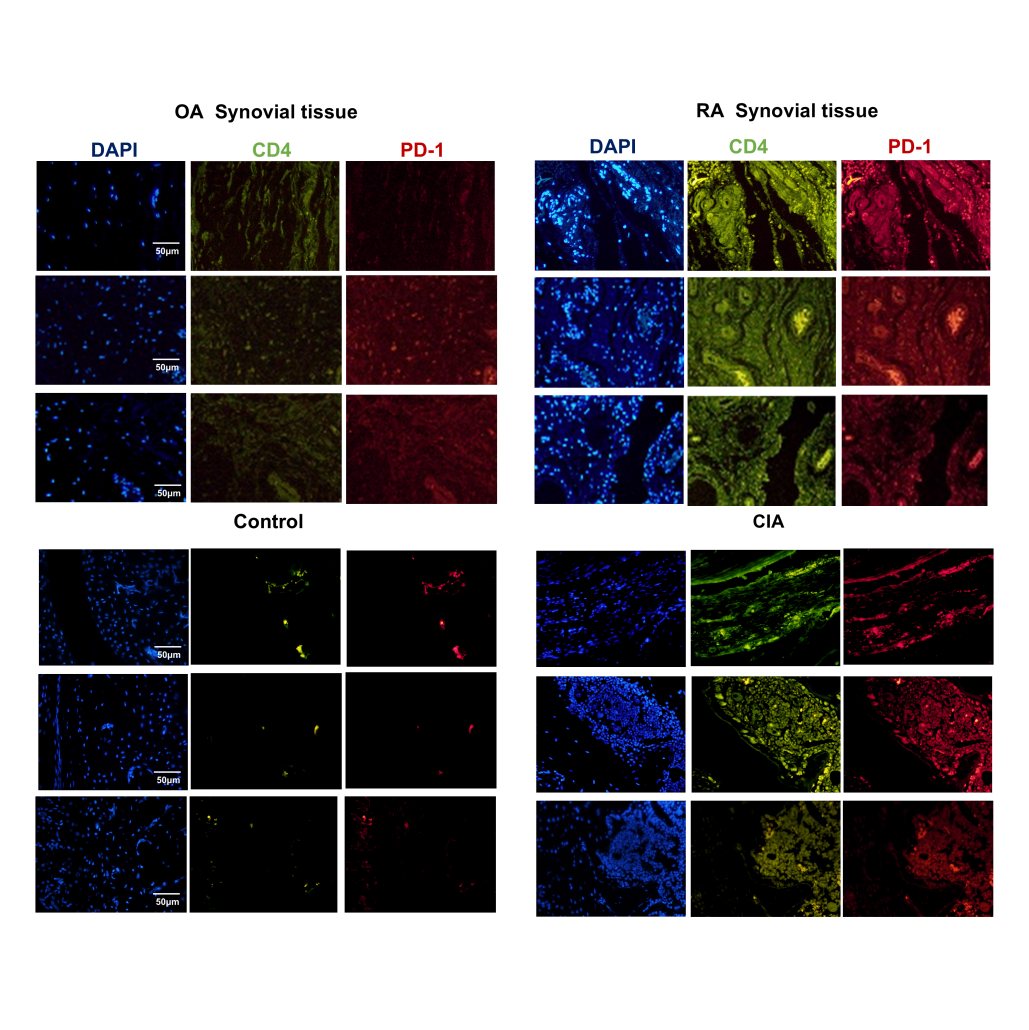


**Figure S3. Immunofluorescent staining of CD4^+^PD-1^+^T cells.** Immunofluorescent staining of CD4^+^PD-1^+^T cells in the synovium of RA patients(n=3), OA patients (n=3), control mice (n=3), and CIA mice (n=3).


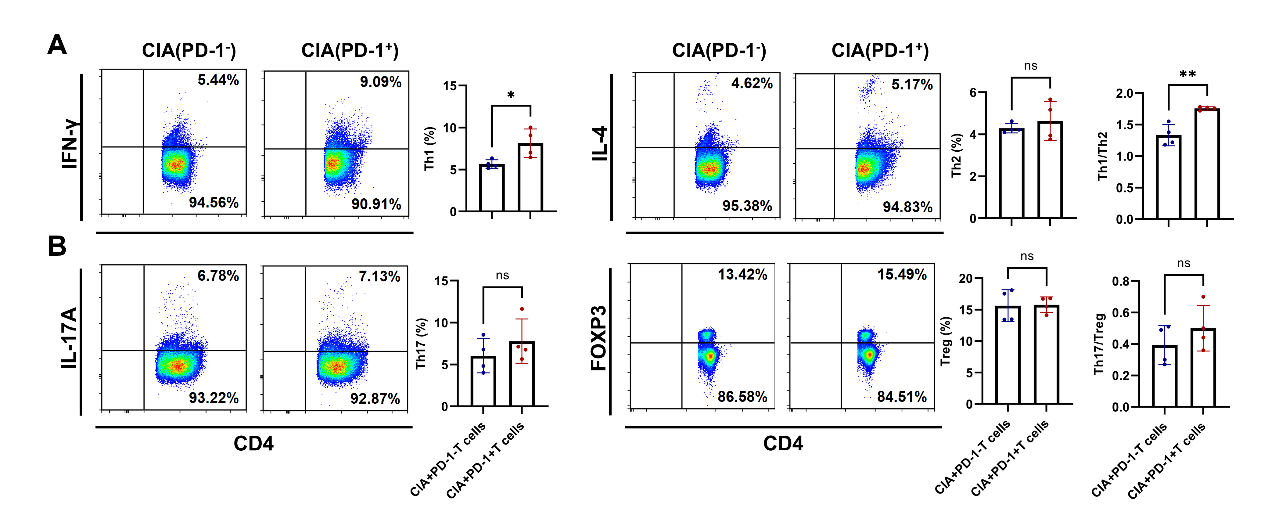


**Figure S4.** **CIA** **CD4^+^PD-1^+^T cell adoptive transfer upregulates the Th1 cell subsets in CIA mice.** CD4^+^PD-1^+^T cells and CD4^+^PD-1^-^T cells were isolated from the spleens of CIA mice, and then respectively adoptively transferred to other CIA mice (n=4). The proportion of Th1 (A), Th2 (B), Th17 (C), and Treg (D) cells in the spleen of CIA mice was analyzed by flow cytometry. Symbols represent individual subjects. ns, no significance; *, *P* < 0.05; **, *P* < 0.01.


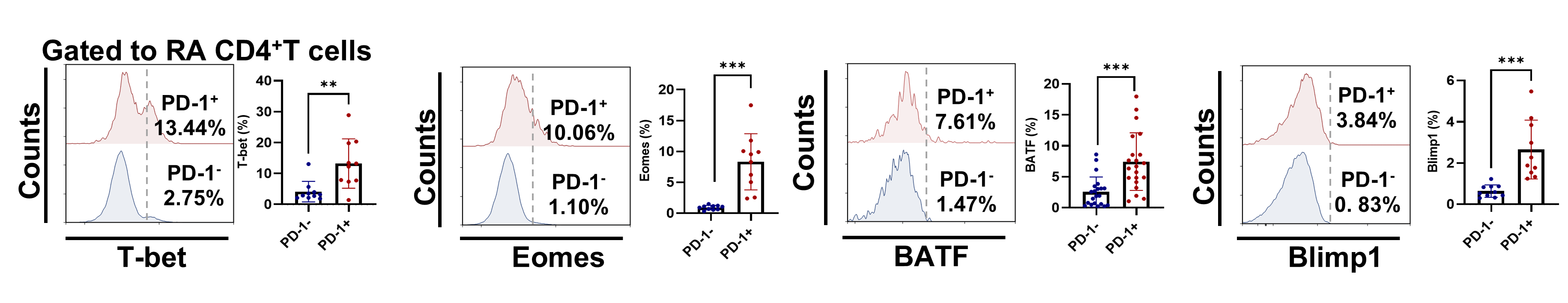


**Figure S5.** **The levels of transcription factors in RA CD4^+^PD-1^+^T cells and CD4^+^PD-1^-^T cells.** The expression of transcription factors (T-bet, n=10; Eomes, n=10; BATF, n=21; Blimp1, n=10) in RA CD4^+^PD-1^+^T cells and CD4^+^PD-1^-^T cells was analyzed by flow cytometry. Symbols represent individual subjects. **, *P* < 0.01; ***, *P* < 0.001.


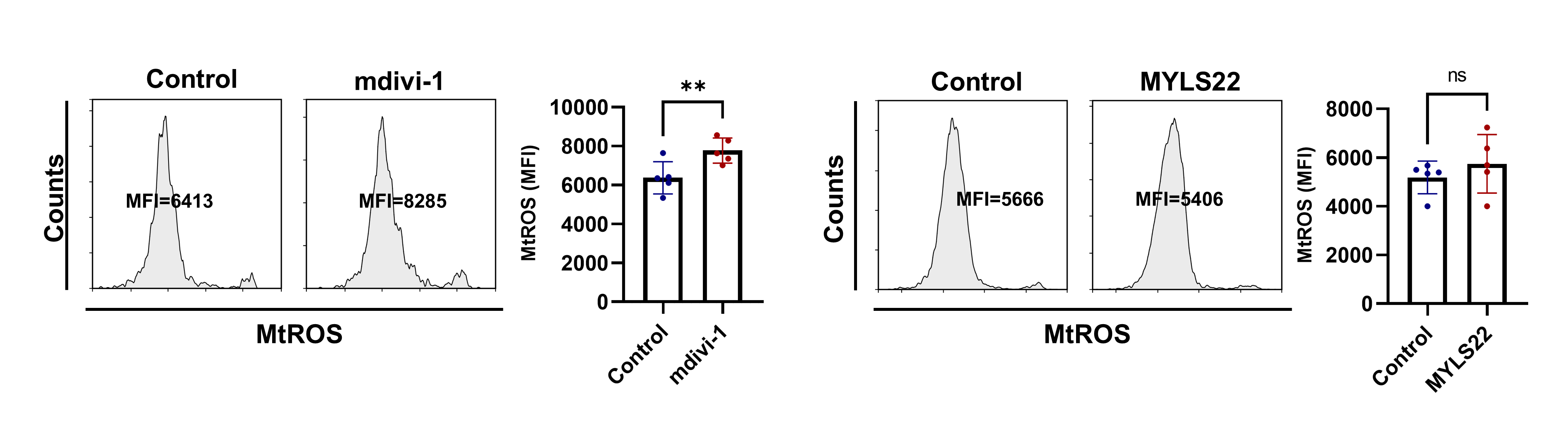


**Figure S6. DRP1 inhibitor upregulated the level of MtROS in RA CD4^+^PD-1^+^T cells.** CD4^+^T cells from RA patients were sorted and treated with DRP1 inhibitor (mdivi-1) and OPA1 inhibitor (MYLS22) for 24 h, and the level of MtROS was analyzed by flow cytometry (n=5). Symbols represent individual subjects. ns, no significance; **, *P* < 0.01.


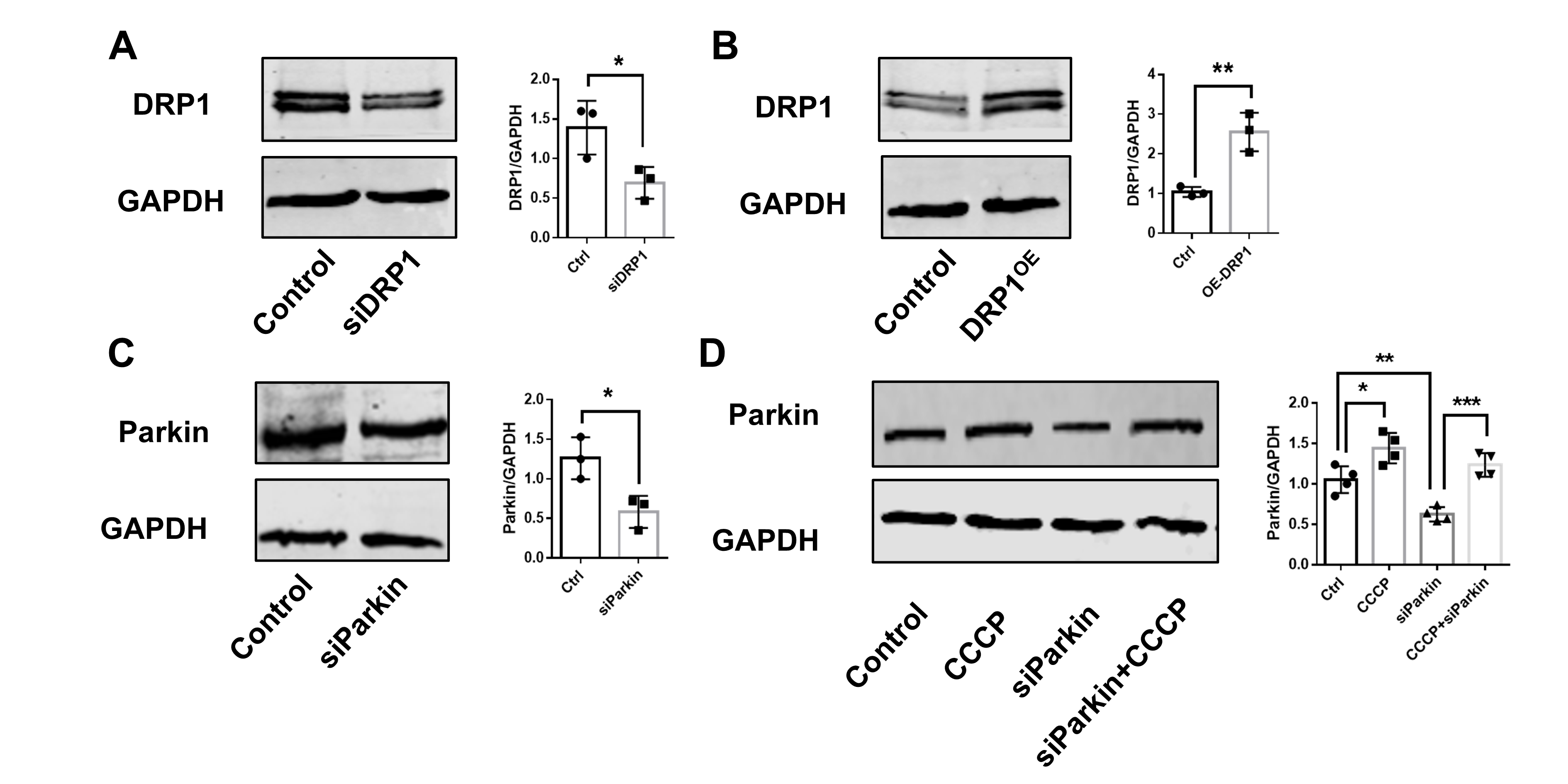


**Figure S7. DRP1 and Parkin Transfection Efficiency and the Effect of CCCP on Parkin.** (A-B) DRP1 expression was analyzed in jurkat cells by Western blot following DRP1 knockdown via siRNA transfection (n=3) or overexpression via plasmid transfection (n=3) in Jurkat cells. (C-D) Parkin expression was analyzed under three conditions: CCCP treatment alone, Parkin knockdown via siParkin transfection, and Parkin knockdown followed by CCCP exposure (n=3 per group).


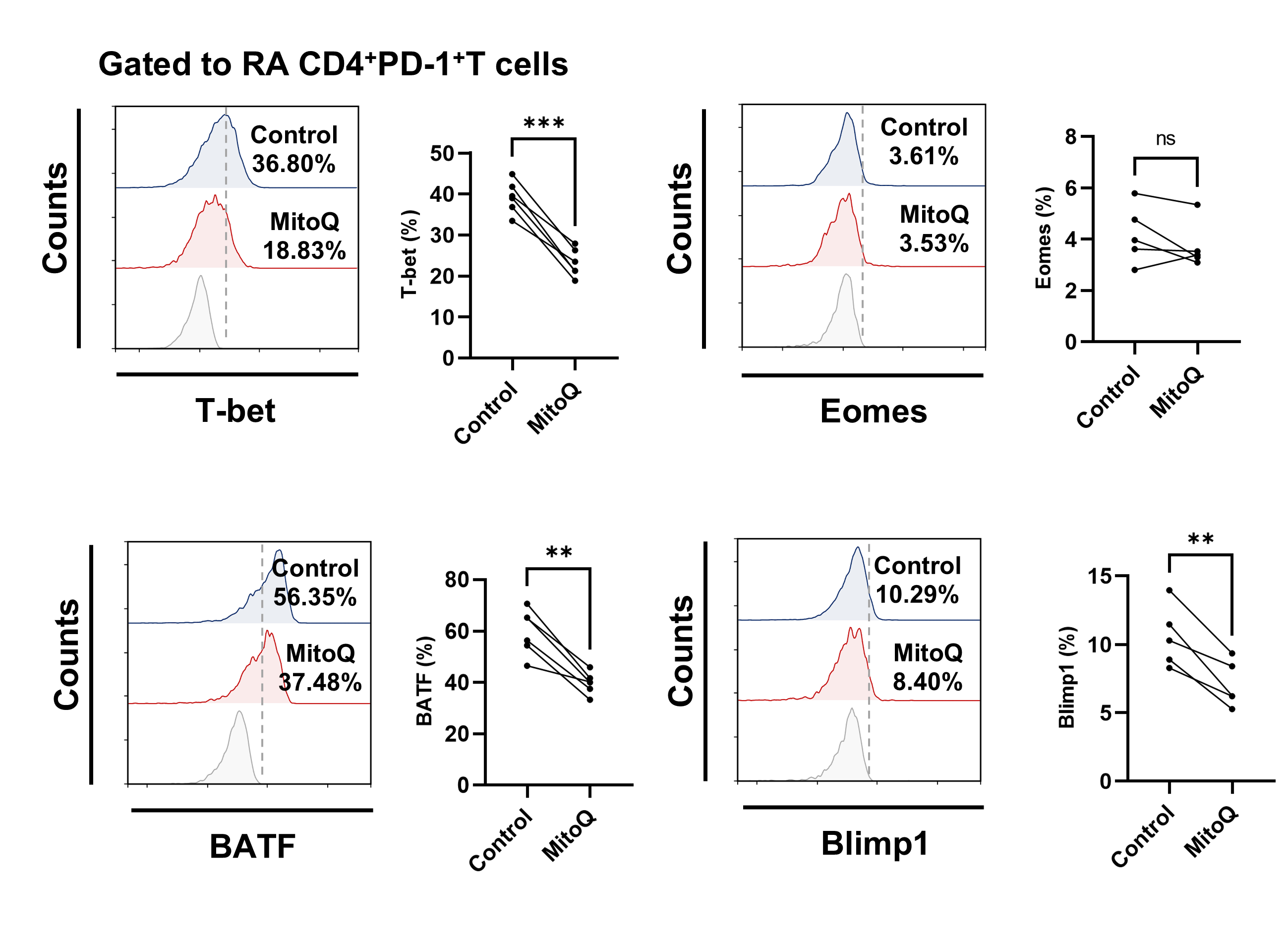


**Figure S8. MtROS scavenging downregulated the expression of transcription factors in RA CD4^+^PD-1^+^T cells.** CD4^+^T cells from RA patients were treated with or without mitoquinone (n=5), and the levels of transcription factors (T-bet, Eomes, BATF, and Blimp1) in CD4^+^PD-1^+^T cells were analyzed by flow cytometry. Symbols represent individual subjects. ns, no significance; **, *P* < 0.01; ***, *P* < 0.001.


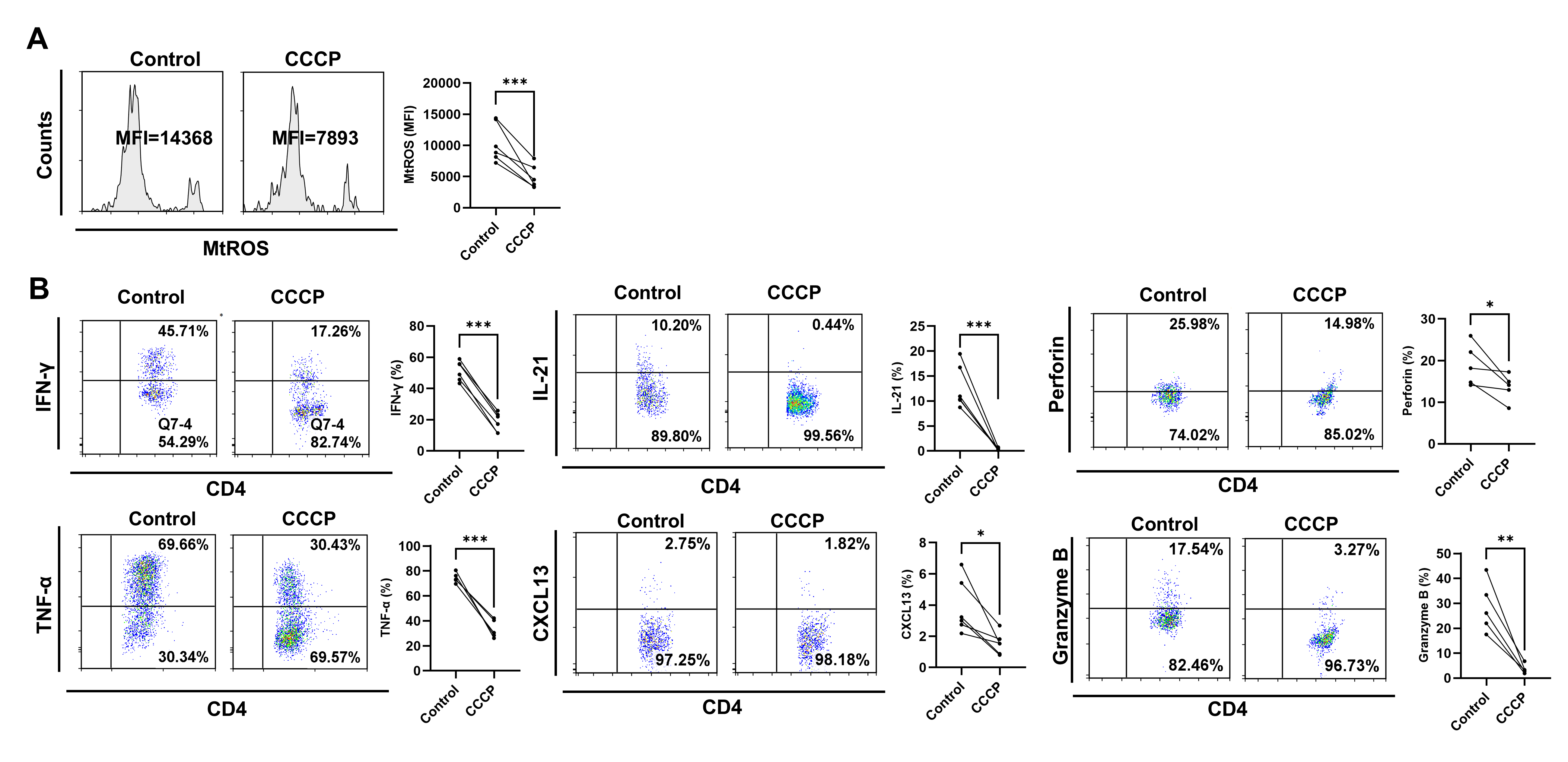


**Figure S9.** **CCCP reduced MtROS and SASP production in RA CD4^+^PD-1^+^T cells.** RA CD4^+^T cells were purified and treated with mitophagy activator (CCCP) for 24-72 h, (A) the MtROS levels (n=6), (B) and the secretion of IFN-γ (n=6), TNF-α (n=5), IL-21 (n=5), CXCL13 (n=6), Perforin (n=5), and Granzyme B (n=5) in RA CD4^+^PD-1^+^T cells were assessed by flow cytometry. Symbols represent individual subjects. *, *P* < 0.05; **, *P* < 0.01; ***, *P* < 0.001.


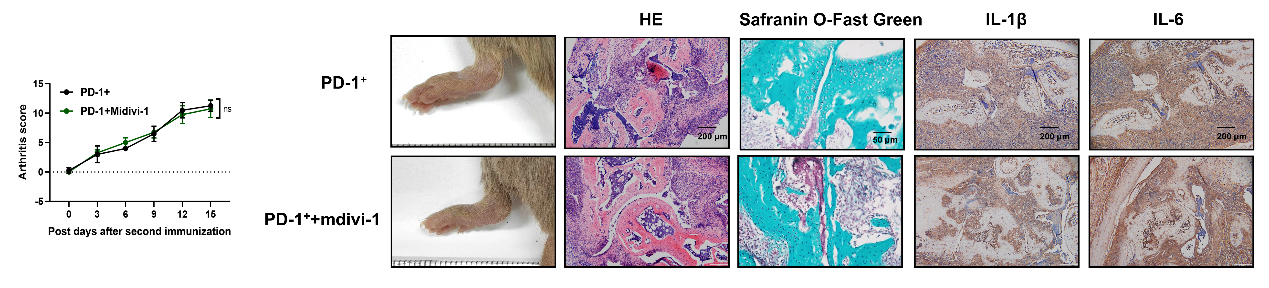


**Figure S10. The joint inflammation of CIA mice that received CD4^+^PD-1^+^T cells and DRP1 inhibitor-treated CD4^+^PD-1^+^T cells.** CD4^+^PD-1^+^T cells were sorted from the spleens of CIA mice, treated with or without mdivi-1 (4 h), and then adoptively transferred into other CIA mice (n=4 per group). The degree of arthritis was observed, and HE staining was used to observe the infiltration of inflammatory cells in the synovium of CIA mice joints. Safranin O-Fast Green staining was used to detect cartilage damage. Immunohistochemical staining was used to detect the levels of IL-1β and IL-6 in the synovium of CIA mice.


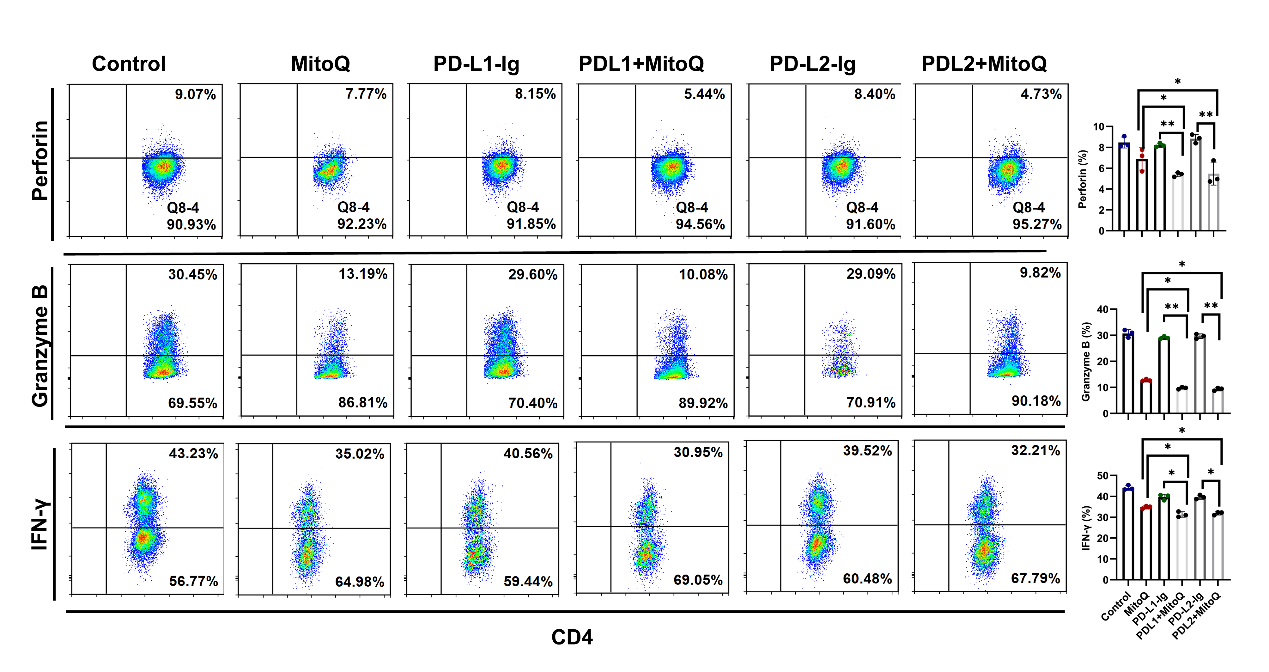


**Figure S11.** **MtROS affects the PD-1 signal in RA CD4^+^T cells.** CD4^+^T cells from RA patients were treated with mitoquinone or PD-1 ligands (PD-L1 or PD-L2) for 72 h, and the levels of Perforin, Granzyme B, and IFN-γ in RA CD4^+^PD-1^+^T cells were analyzed by flow cytometry (n=3). Symbols represent individual subjects. ns, no significance; *, *P* < 0.05; **, *P* < 0.01.


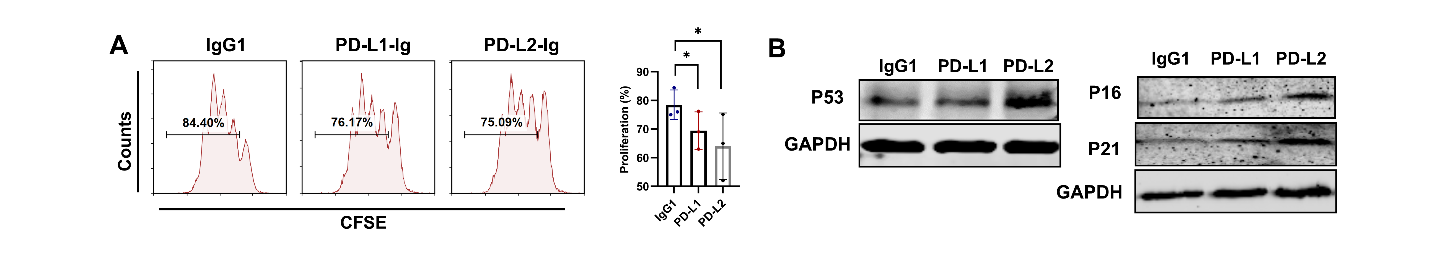


**Figure S12. PD-1 signal inhibited RA CD4^+^PD-1^+^T cell proliferation.** (A-B) CD4^+^T cells from RA patients were sorted and stimulated with IgG1, PD-L1-Ig, or PD-L2-Ig. The proliferation ability (5 days, n=3), the expression of P53, P21, and P16 (48 h, n=3). Symbols represent individual subjects. *, *P* < 0.05.


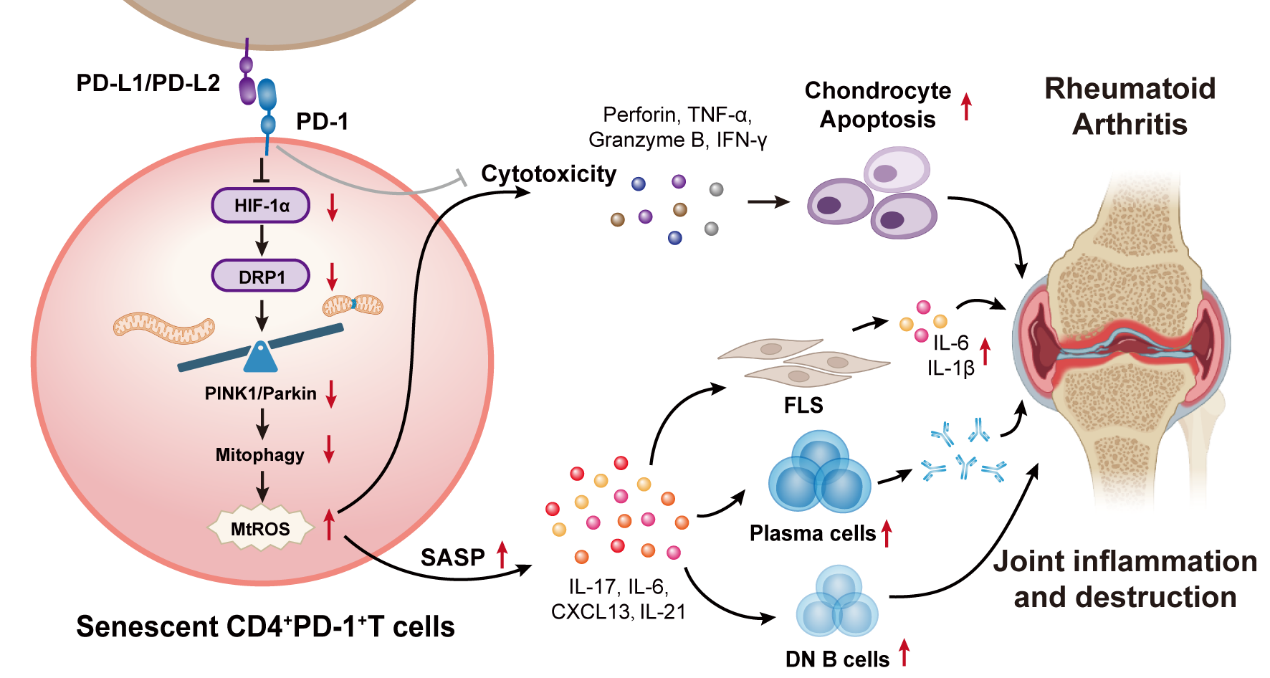


**Figure S13. DRP1 Downregulation Impairs Mitophagy, Driving Mitochondrial ROS and SASP Production in Rheumatoid Arthritis CD4^+^PD-1^+^T Cells.** The downregulation of DRP1 and impaired mitophagy contribute to the increased MtROS production, which in turn promotes the secretion of the SASP and the cytotoxic functions of RA CD4^+^PD-1^+^T cells. Moreover, PD-1 signaling transcriptionally suppressed DRP1 expression through HIF-1α inhibition. DRP1, Dynamin-related protein 1; MtROS, mitochondrial reactive oxygen species; RA, rheumatoid arthritis; HIF-1α: hypoxia inducible factor 1 alpha subunit; FLS: fibroblast-like synoviocytes; DN: double negative; SASP: senescence-associated secretory phenotype.
